# MAPK-driven epithelial cell plasticity drives colorectal cancer therapeutic resistance

**DOI:** 10.1038/s41586-025-09916-w

**Published:** 2025-11-24

**Authors:** Mark White, Megan L. Mills, Laura M. Millett, Kathryn Gilroy, Yourae Hong, Lucas B. Zeiger, Rosalin J. Simpson, Shania M. Corry, Amelia Ligeza, Tamsin R. M. Lannagan, Susanti Susanti, Rachel A. Ridgway, Ayse S. Yazgili, Lucile Grzesiak, Raheleh Amirkhah, Catriona A. Ford, Nikola Vlahov, Hannah Tovell, Leah Officer-Jones, Catherine Ficken, Rachel Pennie, Arafath K. Najumudeen, Alexander Raven, Nadia Nasreddin, Ekansh Chauhan, Andrew S. Papanastasiou, Colin Nixon, Vivienne Morrison, Rene Jackstadt, Janet S. Graham, Crispin J. Miller, Sarah J. Ross, Simon T. Barry, Valeria Pavet, Richard H. Wilson, John Le Quesne, Philip D. Dunne, Sabine Tejpar, Simon Leedham, Andrew D. Campbell, Owen J. Sansom

**Affiliations:** 1 Cancer Research UK Scotland Institute, Glasgow, UK; 2 School of Cancer Sciences, University of Glasgow, Glasgow, UK; 3 Beatson West of Scotland Cancer Centre, Glasgow, UK; 4 Digestive Oncology, Katholieke Universiteit Leuven, Leuven, Belgium; 5 Wellcome Centre for Human Genetics, University of Oxford, Oxford, UK; 6 The Patrick G. Johnston Centre for Cancer Research, Queen’s University Belfast, Belfast, UK; 7 Heidelberg Institute for Stem Cell Technology and Experimental Medicine (HI-STEM gGmbH), Heidelberg, Germany; 8 Germany Cancer Progression and Metastasis Group, German Cancer Research Center (DKFZ), Heidelberg, Germany; 9 OTD Bioscience, Early Oncology, AstraZeneca, Cambridge, UK; 10 Translational Gastroenterology Unit, John Radcliffe Hospital, University of Oxford, Oxford, UK; 11 Oxford NIHR Biomedical Research Centre, Oxford, UK

**Keywords:** Cancer stem cells, Cancer models, Targeted therapies, Cancer therapeutic resistance

## Abstract

The colorectal epithelium is rapidly renewing, with remarkable capacity to regenerate following injury. In colorectal cancer (CRC), this regenerative capacity can be co-opted to drive epithelial plasticity. Although oncogenic MAPK signalling in CRC is common, with frequent mutations of both *KRAS* (40–50%) and *BRAF* (10%)^1^, inhibition of this pathway typically drives resistance clinically. Here, given the development of KRAS inhibitors and licensing of BRAF inhibitor combinations^2–4^, we have interrogated key mechanisms of resistance to these agents in advanced preclinical CRC models. We show that oncogenic MAPK signalling induces epithelial-state changes in vivo, driving adoption of a regenerative/revival stem-like population, whereas inhibition leads to rapid transcriptional remodelling of both *Kras*-mutant and *Braf-*mutant tumours, favouring a WNT-associated, canonical stem phenotype. This drives acute therapeutic resistance in *Kras*-driven and delayed resistance in *Braf*-driven models. Where plasticity is restrained, such as in early metastatic disease, or through targeting ligand-dependent WNT pathway *Rnf43* mutations, marked therapeutic responses are observed. This explains the super response to BRAF + EGFR-targeted therapies previously observed in a BRAF–RNF43 co-mutant patient population, highlighting the criticality of cellular plasticity in therapeutic response. Together, our data provide clear insight into the mechanisms underpinning resistance to MAPK-targeted therapies in CRC. Moreover, strategies that aim to corral stem cell fate, restrict epithelial plasticity or intervene when tumours lack heterogeneity may improve therapeutic efficacy of these agents.

## Abstract (web summary)

In colorectal cancer, therapeutic resistance is driven by MAPK-dependent epithelial cell plasticity.

## Main

Although the key genetic drivers of CRC have been known for decades, recent bulk and single-cell transcriptomic profiling have suggested a small number of distinct CRC subtypes, whose identity cannot be explained directly through mutational burden alone^5–8^. Elegant work in early CRC has suggested two distinct initial tumorigenic routes: either via activated WNT signalling in *LGR5*^+^ canonical stem cells or from *BRAF*-mutated or *KRAS*-mutated serrated lesions enriched for metaplastic and regenerative markers^8^. These are also evident in established CRCs, described as iCMS2 (WNT-high) or iCMS3 (regenerative/metaplastic) cells^7^. Under homeostatic conditions, the canonical crypt-base stem cell population is marked by *LGR5*, whereas damage or injury can induce regeneration through a multipotent *LGR5*^*−*^ population^9–12^. This plasticity is retained in tumours and can influence progression, metastasis and responses to chemotherapy^13–16^. Mutations set the ‘tone’ of the predominant epithelial stem fate with *APC* mutations associated with a canonical, *LGR5*^+^, WNT-enriched population and MAPK mutations with the regenerative fate^17^. Critically, tumours contain both populations, regardless of somatic mutation. Finally, single-cell transcriptional profiling of normal colon, alongside matched primary and metastatic tumours showed that tumour epithelial plasticity can be influenced by external factors^16^. Consequently, epithelial cellular plasticity has been hypothesized as a mode of therapeutic resistance in CRC.

Deploying a suite of in vivo models of CRC, we demonstrated that *Kras*-driven or *Braf*-driven MAPK activation enriches for cell populations marked by expression of regeneration-associated genes. Meanwhile, nascent liver metastases are transcriptionally restricted to a single cellular state, with heterogeneity established in later disease, inducing early vulnerability to targeted MAPK inhibition. Finally, *Kras*-targeted or *Braf*-targeted inhibition in CRC drives epithelial cell fate towards a WNT-high state, with *Rnf43* loss sensitizing to MAPK inhibition in *Braf*-mutant disease through restraint of epithelial plasticity. Therefore, MAPK-targeting therapies induce tumour epithelial-state changes, leading to maintained tumour viability, and contributing to heterogeneity in upfront responses, and rapidly emergent resistance to MAPK therapies.

## Mutation influences epithelial identity

**Fig. 1:**
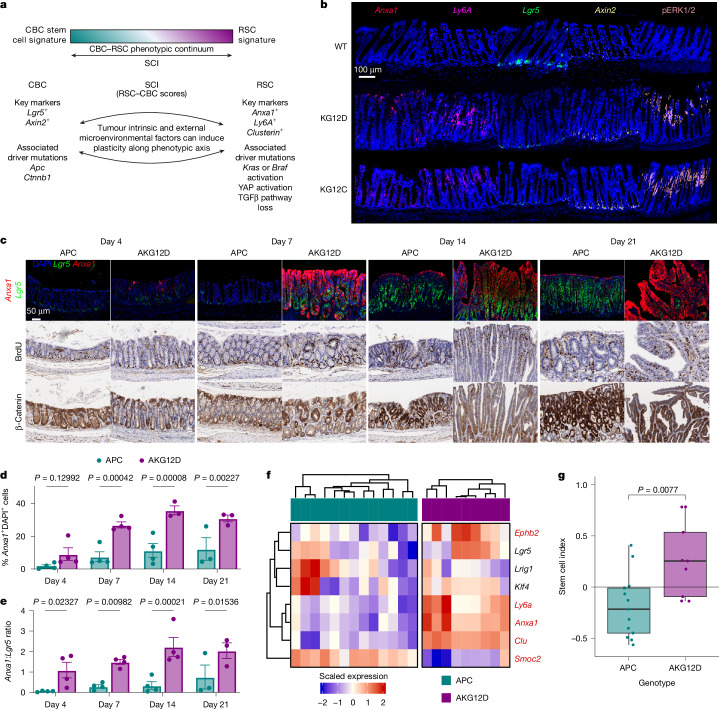
KRAS activation drives an RSC phenotype. **a**, Schema representing CRC cell states, associated key markers and driver mutations. Cells can adopt a CBC state, a RSC state or exist on a continuum between these states. The SCI is a composite measure of these, describing the phenotypic continuum between states. Data adapted from ref. ^13^. The diagram was created in BioRender. White, M. (2025) https://BioRender.com/3s904bg. **b**, Representative images of *Anxa1*, *Ly6a* (RSC markers), *Lgr5*, *Axin2* (CBC markers) fluorescence in situ hybridization (FISH) and pERK IHC in colons of VillinCre^ERT2^ (WT), KG12D and KG12C. The representative images of mouse colonic tissue were sampled 30 days post-intraperitoneal tamoxifen induction (DPI) (*n* = 5 for WT, *n* = 3 for KG12D and *n* = 6 for KG12C). **c**, Time course of APC and AKG12D following intracolonic tamoxifen injection 4, 7, 14 and 21 DPI. Representative images of serial sections of β-catenin and BrdU IHC and with *Anxa1* (red) and *Lgr5* (green) FISH. For BrdU IHC, *n* = 3 (days 14 and 21) and *n* = 4 (days 4 and 7) for APC, *n* = 3 (days 7, 14 and 21) and *n* = 4 (day 4) for AKG12D. For β-catenin IHC and *Anxa1*/*Lgr5* FISH analyses, *n* = 3 (day 21) and *n* = 4 (days 4, 7 and 14) for APC, *n* = 4 (days 4 and 7) and *n* = 3 (days 14 and 21) for AKG12D. **d**,**e**, Proportion of *Anxa1*^+^DAPI^+^ cells (**d**) and *Anxa1*^*+*^:*Lgr5*^*+*^ ratio (**e**) in the time course of APC and AKG12D at 4, 7, 14 and 21 DPI. Significance was determined by two-tailed Student’s *t*-tests, no multiple comparison correction. Data are mean ± s.e.m. **f**, Scaled heatmap of normalized gene expression associated with the CBC or RSC phenotype by RNA-seq of intracolonic tumours from APC (*n* = 13) and AKG12D (*n* = 9) mice. Statistical testing was performed by two-sided Wald test; the gene symbols in red have a Benjamini–Hochberg adjusted *P* < 0.05. **g**, SCI determined from RNA-seq of intracolonic tumours from APC (*n* = 13) or AKG12D (*n* = 9) mice. Boxes are median and interquartile range (IQR), and whiskers extend to the minimum and maximum values reaching up to 1.5× the lower and upper IQR. Significance was determined using a two-tailed Student’s *t*-test.

Stem-like epithelial populations are commonly reported in CRC, with subtleties in description and nomenclature, but sharing consistent features^17,18^. These include the presence of homeostatic and regenerative stem-like populations, a relationship between oncogenic mutation and cellular identity, and clear tumour epithelial heterogeneity. We have previously identified epithelial cell fates defined by stem-related signatures across human and mouse CRC datasets^13^, defined by canonical, crypt-base columnar (CBC) or regenerative stem cell (RSC) signatures, featuring elevated *Lgr5* expression or enrichment of the fetal intestinal markers *Anxa1* and *Ly6a* (SCA1), respectively (Fig. 1a). Relative enrichment of RSC and CBC signatures was used to assign samples with a ‘stem-cell index’ (SCI), describing their epithelial phenotype. Integrative analysis of mouse and human single-cell RNA sequencing (scRNA-seq) data suggests that epithelial cell fates can be described in terms of key oncogenic pathways^19^, which align closely to these two key cell fates.

**Extended Data Fig. 1:**
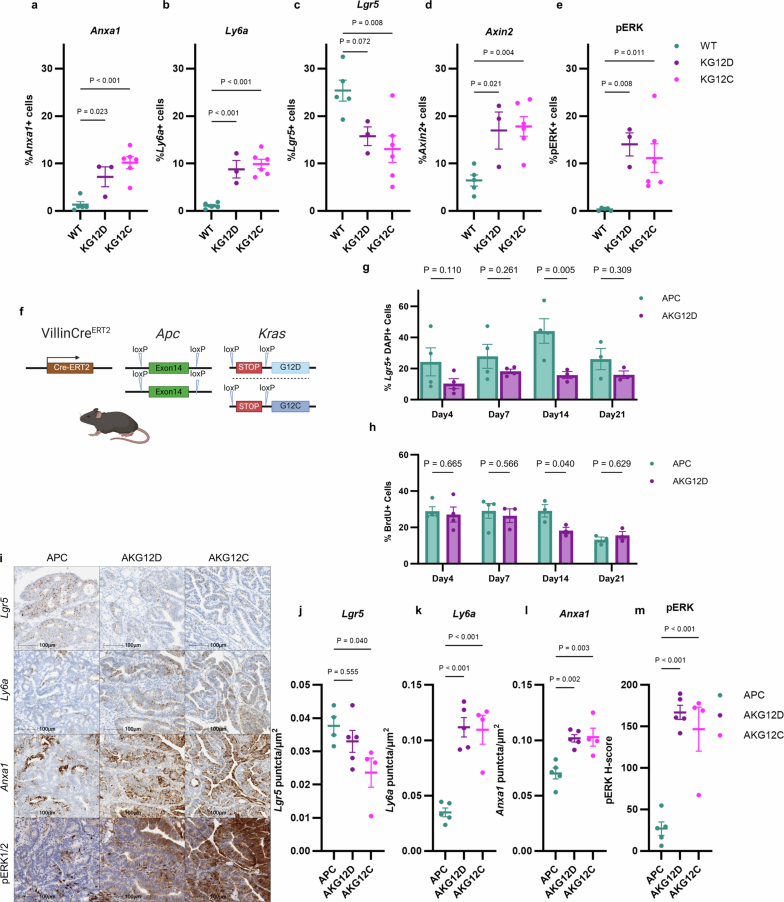
KRAS activation drives RSC marker expression in colonic tissue and tumours. A-E Quantification of indicated stains of colonic tissue from Fig. 1b across VillinCre^ERT2^ (WT), KG12D and KG12C 30 days post tamoxifen induction. Mean ± SEM, One-way ANOVA with Dunnett’s correction for multiple comparisons. WT n = 5, KG12D n = 3, KG12C n = 6. F- Schematic description of genetic crossing strategies to generate APC, AKG12D and AKG12C mice. The schematic was created in BioRender. White, M. (2025) https://BioRender.com/c0vov8d. G-H- Quantification of percentage of *Lgr5*^+^ DAPI^+^ cells and BrdU positive cells in recombined tissue of time-course sampling of APC and AKG12D at intracolonic tamoxifen injection site 4,7,14 and 21 days post induction. For Lgr5+ DAPI+ stain, APC n = 3 (day 21) and n = 4 (days 4, 7 and 14). AKG12D n = 4 (days 4 and 7) n = 3 (days 14 and 21). For BrdU+ stain, APC n = 3 (days 14 and 21) and n = 4 (days 4 and 7). AKG12D n = 3 (days 7, 14 and 21) and n = 4 (day 4). Mean ± SEM. Two-tailed t-tests, no correction for multiple comparisons. I- Representative images for annotated stains of colonic tumours from APC, AKG12D, AKG12C mice, induced with intracolonic 4-hydroxytamoxifen (4-OHT). J-M- Quantification of indicated stains of APC (n = 4 and n = 5), AKG12D (n = 5) and AKG12C (n = 4) colonic tumours. Mean ± SEM, One-way ANOVA with Dunnett’s correction for multiple comparisons.

*Kras* mutations are common in CRC, with activated oncogenic *Kras* reported to drive epithelial proliferation and altered differentiation^20,21^. Given the association between MAPK activation and the RSC, we investigated the effect of oncogenic KRAS(G12D) and KRAS(G12C) mutations on epithelial identity in vivo using Cre-Lox technology. Colonic tissues from VillinCre^ERT2^ *Kras*^*G12D/+*^ (KG12D) and VillinCre^ERT2^ *Kras*^*G12C/+*^ (KG12C), were examined for expression of *Lgr5*, *Anxa1* and *Ly6a* as markers of epithelial cell state, with *Axin2* and ERK1/2 phosphorylation to report WNT and MAPK activation, respectively. These samples were characterized by increased MAPK activation compared with wild-type (WT) colon tissue, concomitant with expression of the RSC markers *Anxa1* and *Ly6a*, and reduced expression of the CBC marker *Lgr5* (Fig. 1b and Extended Data Fig. 1a–e). Thus, oncogenic MAPK activation skews intestinal epithelial tissue towards the RSC cell fate in vivo.

Next, the effect of *Kras* mutation on epithelial identity in the context of APC loss was assessed, as APC loss drives unrestrained WNT signalling, supporting transcription of the CBC gene signature, whereas *Kras* mutation is associated with adoption of the regenerative, RSC gene signature. Colonic tumorigenesis was driven by localized genetic recombination in the colonic epithelium of VillinCre^ERT2^ *Apc*^*fl/fl*^ (APC) and VillinCre^ERT2^ *Apc*^*fl/fl*^*Kras*^*G12D/+*^ (AKG12D; Extended Data Fig. 1f) mice, with subsequent sampling at 4, 7, 14 and 21 days post-induction (DPI). There was marked rapid induction of *Anxa1* in AKG12D as early as 4 DPI (Fig. 1c,d), with this adoption of a regenerative cell fate occurring despite the presence of nuclear β-catenin, typically associated with a CBC-like transcriptional state. The CBC marker *Lgr5* was the key feature of APC tumorigenesis at all stages, and although also observed in AKG12D, these tumours predominantly exhibited expansion of the RSC population over time (Fig. 1e and Extended Data Fig. 1g). Cellular proliferation was comparable between genotypes (Extended Data Fig. 1h), indicating that compounding oncogenic *Kras* mutation promotes the initiation, expansion and maintenance of the RSC state rather than driving tumour expansion through increased proliferation.

**Extended Data Fig. 2:**
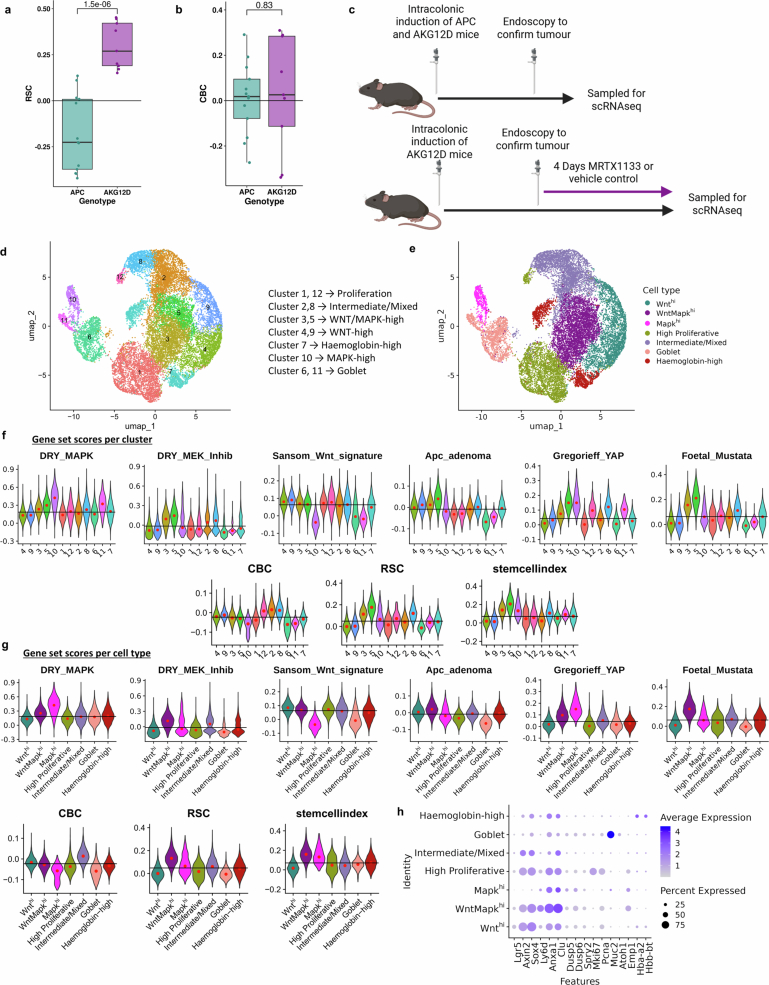
Epithelial cell phenotyping in colon tumours from APC and AKG12D with or without KRAS G12D inhibition. A-B- Regenerative stem cells (RSC) and crypt base columnar (CBC) scores from bulk RNA sequencing of intracolonic tumours from APC (n = 13) or AKG12D (n = 9) mice. Boxes are median and IQR, whiskers extend to minimum and maximum values reaching up to 1.5*lower and upper IQR. Two-tailed t-test. C- Schematic description of single cell RNA sequencing series experiment of APC, AKG12D, AKG12D mice treated with vehicle and AKG12D mice treated with MRTX1133 (KRAS-G12D inhibitor). The schematic was created in BioRender. White, M. (2025) https://BioRender.com/c02uw1d. D-E- UMAP visualisation of epithelial cells from scRNAseq of colonic tumours from APC mice (2,442 cells from 4 biological replicates), AKG12D mice (4439 cells from 3 biological replicates), AKG12D mice treated with vehicle (5,429 cells from 3 biological replicates) and AKG12D mice treated with MRTX1133 (5,823 cells from 5 biological replicates). Original 12 clusters (D) and the 7 key cell states (E) alongside the cluster numbers that make up the 7 key states. F-G- Violin plots of module scores of MAPK pathway activation (“Dry_MAPK” and “Dry_MEK_Inhib”), WNT pathway activation (“Apc_adenoma” and “Sansom_Wnt_signature”), YAP/Foetal (“Gregorieff_YAP” and “Foetal_Mustata”), crypt base columnar (CBC) scores, regenerative stem cell (RSC) scores and stem cell index (SCI) across 12 clusters (C) and the 7 key cell states (D). Horizontal line represents median of all the module scores. H- Key individual marker genes relating to epithelial cell state across cell populations. Dot size represents percentage of expressing cells and colour indicates average expression.

Given the adoption of the RSC in early tumorigenesis, we assessed whether this was retained in established tumours. Established colonic tumours from the AKG12D and related VillinCre^ERT2^ *Apc*^*fl/fl*^*Kras*^*G12C/+*^ (AKG12C) model exhibited expression of RSC markers *Anxa1* and *Ly6a*, and restricted CBC marker *Lgr5* (Extended Data Fig. 1i–m). Transcriptomic analysis of these tumours highlighted enrichment of RSC-associated biomarkers *Ly6a*, *Clu* and *Anxa1* in AKG12D tumours, concomitant with a reduction in the CBC marker *Smoc2* (Fig. 1f). CBC, RSC and SCI transcriptional scores^13^ highlighted induction of RSC and, consequently, elevation of SCI in response to KRAS activation (Fig. 1g and Extended Data Fig. 2a,b). Overall, KRAS activation promotes the RSC phenotype even in the context of APC mutation.

## Tumours comprise many epithelial fates

To determine whether KRAS mutation drove global upregulation of an RSC-related transcriptional program or expansion of individual epithelial subpopulations, we profiled APC and AKG12D colonic tumours with single-cell resolution. Contemporaneously, AKG12D tumours from mice treated with either vehicle or KRAS(G12D) inhibitor (MRTX1133) were profiled to understand the impact of therapeutic pressure (Extended Data Fig. 2c).

**Fig. 2:**
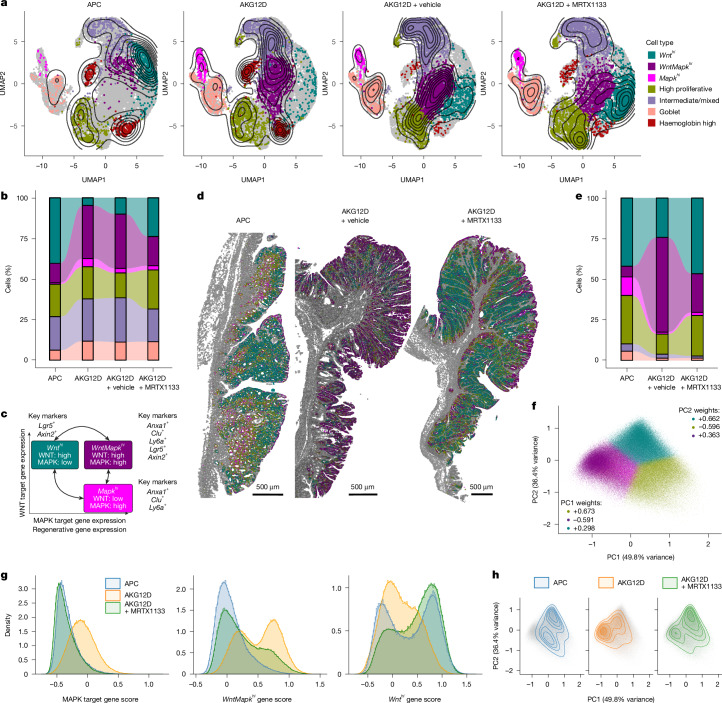
KRAS inhibition shifts epithelial phenotype. **a**, Uniform manifold approximation and projection (UMAP) visualization of epithelial cells and major cell-fate clusters with density overlay mapping from scRNA-seq of colonic tumours from APC (2,442 cells, *n* = 4), AKG12D (4,439 cells, *n* = 3), AKG12D mice + vehicle (5,429 cells, *n* = 3) and AKG12D mice + MRTX1133 (5,823 cells, *n* = 5). **b**, Alluvial plot showing the proportion of cell states across experimental conditions in panel **a**. **c**, Schema summary of key epithelial cell states in CRC derived from panel **a** based on oncogenic signalling pathways and key marker genes for each state. The *x* axis represents increasing expression of genes relating to MAPK activation and regenerative programmes, and the *y* axis represents expression of WNT pathway targets. The schematic was created in BioRender. White, M. (2025) https://BioRender.com/lxn2xfr. **d**, Representative images of spatial distribution annotated cell states in Xenium in situ datasets for APC or AKG12D tumours treated with vehicle or MRTX1133 for 4 days (*n* = 3 tumours per condition). Non-tumour epithelial cells are in grey. **e**, Alluvial plot showing the proportion of cell states across experimental conditions from spatial transcriptomic data of panel **d**. **f**, PCA of cell-type probability from spatial transcriptomic data of panel **d** for the six tumour cell populations generated from scRNA-seq. PC1 describes 49.8% of variance and the top three loadings are high proliferative, *WntMapk*^*hi*^ and *Wnt*^*hi*^. PC2 describes 36.4% of variance and the top three loadings are *Wnt*^*hi*^, high proliferative and *Mapk*^*hi*^. The cell colour denotes the highest probability cell-type call. **g**, Histogram of gene set scores for canonical MAPK target genes, *WntMapk*^*hi*^ and *Wnt*^*hi*^ in tumour epithelial cells of spatial transcriptomic data of panel **d**. The *x* axis is gene score, and the *y* axis is cell density (total cells per condition normalized to an area under the curve of 1). Blue denotes APC (76,475 cells), yellow indicates AKG12D + vehicle (153,013 cells) and green shows AKG12D + MRTX1133 (150,894 cells). **h**, Tumour epithelial cells projected on the PCA space from panel **f**. Blue denotes APC, yellow indicates AKG12D + vehicle and green shows AKG12D + MRTX1133.

The tumour epithelium comprised 12 Leiden clusters, which were assigned to 7 cell fates based on transcriptional module score (Extended Data Fig. 2d–f). Of these, three populations were distinguishable by features related to the CBC–RSC axis, namely, WNT or MAPK activation/regenerative stem marker expression. Additional populations representing highly proliferative, goblet-like, intermediate/mixed and haemoglobin gene profiles were also observed. Each cell type was robustly represented in all groups, with significant variation in proportion based on genotype and treatment (Fig. 2a,b). Here we observed a population exhibiting enrichment of the CBC signature-enriched population, with suppressed RSC as expected, but two distinct populations driven by the RSC signature, differentiated by relative enrichment of CBC. As the CBC-enriched cell fate here was driven by WNT-dependent transcriptional programmes and expression of key WNT-driven marker genes, it is henceforth described as a ‘*Wnt*^*hi*^’ population. Similarly, the RSC-enriched cell populations were driven by MAPK-associated programmes, and could be differentiated by WNT pathway activation, and thus these are henceforth described as ‘*Mapk*^*hi*^’ and ‘*WntMapk*^*hi*^’ (Fig. 2c and Extended Data Fig. 2g,h). When compared with APC tumours, AKG12D exhibited reduced *Wnt*^*hi*^ and increased *WntMapk*^*hi*^ populations, with no effect observed on other cell fates. The proportion of highly proliferative cells was unchanged, reinforcing the lack of pro-proliferative effect of KRAS mutation in this setting. Therefore, tumours are transcriptionally heterogeneous, irrespective of driver mutation, with the major effect of the mutation being an alteration of the relative proportion of each individual cell population.

## Therapy influences tumour cell identity

**Extended Data Fig. 3:**
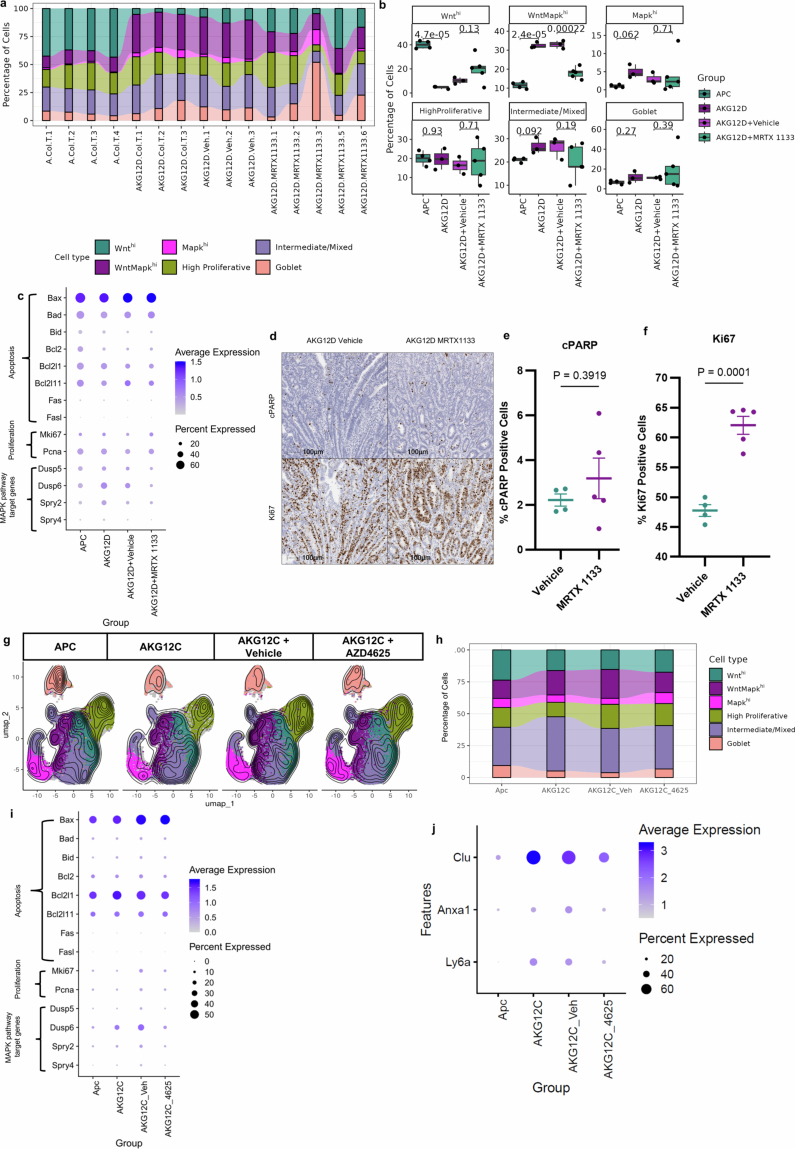
Epithelial cellular plasticity in response to KRAS inhibition. A- Bar graph showing major epithelial populations across experimental conditions form scRNAseq associated with Fig. 2a,b at an individual sample level B- Boxplots comparing the cell type proportion of four experimental conditions. Each dot represents the cell type proportion of each sample. Colours indicated each experimental condition. Two-tailed t-test. Boxes are median and interquartile ranges (IQR) with whiskers extending from IQR to minimum and maximum values up to 1.5 * lower and upper IQR respectively. C- Individual marker genes relating to apoptosis, proliferation and MAPK-pathway signalling across each experimental condition from scRNAseq. Dot size represents percentage of expressing cells and colour indicates average expression. D- Representative images of AKG12D colonic tumours following 4-day treatment with vehicle or MRTX1133. IHC images of the apoptotic marker cleaved PARP (cPARP) or proliferation (Ki67) are presented. E- Quantification of percentage cPARP positive cells in colonic tumours of AKG12D mice treated with vehicle (n = 4) or MRTX1133 (n = 5) for 4 days. Mean ± SEM. Two-tailed t-test. F- Quantification of percentage Ki67 positive cells in colonic tumours of AKG12D mice treated with vehicle (n = 4) or MRTX1133 (n = 5) for 4 days. Mean ± SEM. Two-tailed t-test. G- UMAP visualisation of epithelial cells and major cell fate clusters from single cell RNA sequencing of colonic tumours from APC mice (30980 cells from 4 biological replicates), AKG12C mice (11821 cells from 2 biological replicates), AKG12C mice treated with vehicle (41963 cells from 4 biological replicates) or AZD4625 (33527 cells from 5 biological replicates). H- Alluvial bar graph showing percentage of cells of major epithelial populations across experimental conditions from scRNAseq in Extended Data Fig. 3g. I-J Individual marker genes relating to apoptosis, proliferation and MAPK-pathway signalling across each experimental condition in scRNAseq data from Extended Data Fig. 3g. Dot size represents percentage of expressing cells and colour indicates average expression.

Given the phenotypic effect of KRAS(G12D) mutations, we next investigated the effect of therapeutic pressure on cell fate. Tumour-bearing AKG12D mice were treated with MRTX1133, resulting in restriction of the *WntMapk*^*hi*^ population and enrichment of the *Wnt*^*hi*^ population, producing a cell-fate distribution similar to an APC tumour (Fig. 2b and Extended Data Fig. 3a,b). This occurred after only 4 days of treatment, suggesting that epithelial plasticity is an intrinsic adaptive response to treatment, rather than a process of acquired resistance. Again, despite a reduction of canonical MAPK pathway target genes (*Dusp5*, *Dusp6* and *Spry2*, *Spry4*) suggesting efficient MAPK suppression, the highly proliferative cell population was unaffected by treatment, indicating a lack of any cytostatic effect, and there was no induction of apoptotic markers across treated datasets (Extended Data Fig. 3c). In line with transcription, treatment had no effect on the abundance of cleaved PARP, whereas the proportion of Ki67-positive proliferative cells was increased, again suggesting an intrinsic adaptation to inhibitor treatment (Extended Data Fig. 3d–f). Therefore, oncogenic KRAS mutation and subsequent inhibition shifts epithelial cell fate along a ‘phenotypic continuum’ as a therapeutic resistance mechanism. Consequently, rather than any antitumour effect, manipulating this cellular adaptive epithelial plasticity may be the key impact of MAPK inhibitors in CRC.

Although KRAS(G12D) is among the most common mutations found in CRC, other KRAS mutations also occur. We therefore examined scRNA-seq datasets comprising colonic tumours from untreated APC and AKG12C mice, alongside AKG12C mice treated with either vehicle or AZD4625 (KRAS(G12C) inhibitor; Extended Data Fig. 3g,h). Here the KRAS(G12C) mutation drove increased MAPK activation, reported by *Dusp6* expression, and induction of RSC markers *Clu*,* Anxa1* and *Ly6A*, which was reversed following treatment (Extended Data Fig. 3i,j). Despite single-gene changes, the effect on epithelial state was less pronounced than AKG12D tumours, namely, a non-significant increase in the *WntMapk*^*hi*^ population, again lost upon treatment. The differential effect of KRAS(G12D) versus KRAS(G12C) is unsurprising, with specific KRAS mutations displaying different biochemical properties, with KRAS(G12C) having higher rates of intrinsic GTP hydrolysis, increasing time spent in the off-state.

## Therapeutic impact visualized spatially

**Extended Data Fig. 4:**
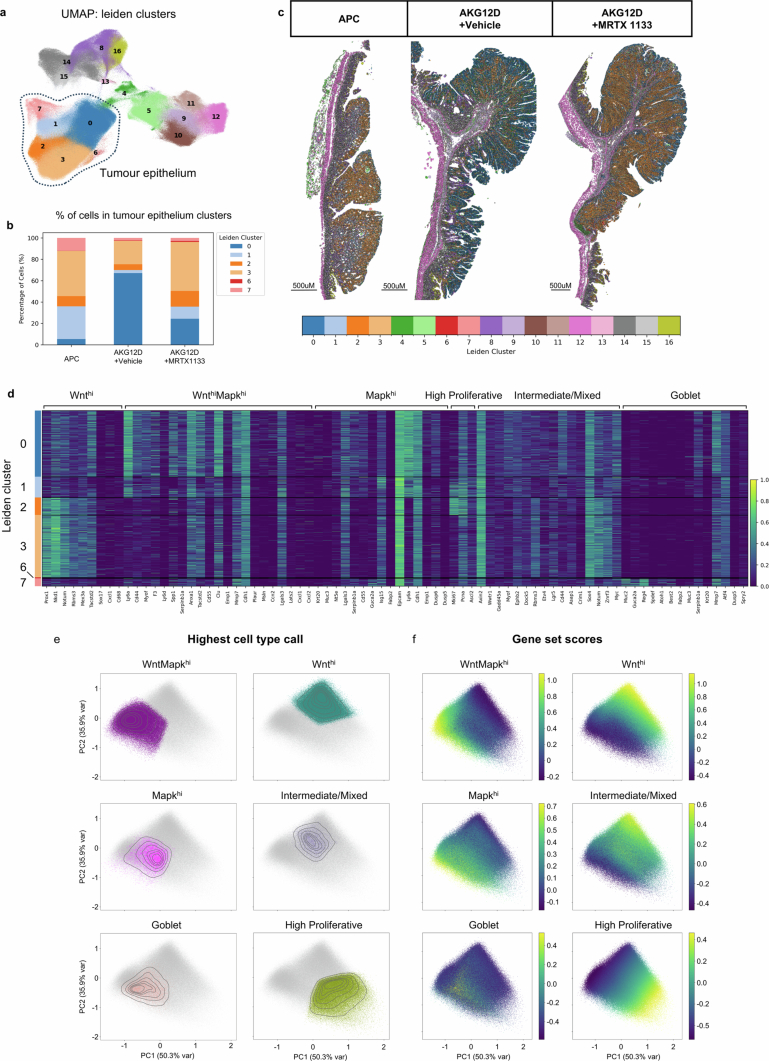
Spatial transcriptomics show epithelial plasticity with KRAS G12D inhibition. A- UMAP projection of APC, AKG12D mice treated with vehicle or MRTX1133 (n = 3 for each condition) cells after 10X Xenium spatial transcriptional profiling. Cells are coloured based on Leiden clustering. Tumour epithelial clusters were identified based on their spatial distribution in the tissue and key marker gene expression. B- Stacked bar graph of Leiden cluster assignment of tumour epithelial cells grouped by their experimental condition (n = 3 for each). C- Representative images of spatial distribution of all cells coloured by their Leiden cluster association (n = 3 for each). Scale 500um. D- Heatmap showing alignment of Leiden clusters presented in Extended Data Fig. 4a to key marker genes of cell populations from scRNAseq data. Colour bar is gene expression column min/max. E- PCA from Fig. 2f showing highest cell type call in this space. Cells are coloured by their highest probability for cell type assignment. F- Gene set scores for the individual gene sets overlayed on PCA presented in Fig. 2f.

To spatially resolve transcriptomic findings, we analysed tumours from the APC, AKG12D vehicle and AKG12D MRTX1133-treated groups using Xenium in situ imaging. We performed Leiden clustering to identify cell populations, including the tumour epithelium (Extended Data Fig. 4a). Cluster 0 cells, expressing high levels of RSC-related markers, were enriched in AKG12D vehicle-treated tumours, whereas clusters 2 and 3, expressing CBC and WNT pathway-related genes, were enriched in APC and AKG12D MRTX1133-treated tumours (Extended Data Fig. 4b–d). To align spatial transcriptomics to earlier data, differential gene expression analysis was performed on scRNA-seq comparison of APC and AKG12D tumours with or without treatment, identifying genes whose expression was enriched in each epithelial cell population. Differentially expressed genes represented in the in situ gene panel were used to assign cellular identity for spatial analysis. This approach provided a remarkable overlap with scRNA-seq analyses. All three experimental groups exhibited heterogeneous epithelial populations, with KRAS mutation driving enrichment of a *WntMapk*^*hi*^ population, counteracted by MRTX1133 treatment (Fig. 2d,e). The residual post-treatment *WntMapk*^*hi*^ population appears restricted to the lumen-exposed surface, with the tumour core predominantly comprising *Wnt*^*hi*^ cells. The morphology and tissue architecture of AKG12D tumours were not altered with treatment, again highlighting that the effect of KRAS inhibition is restricted to cellular plasticity without affecting gross tumour organization.

Tumour epithelial cells express mixtures of key cellular fate signatures. To investigate this further, we assigned each cell a probability of belonging to each cell fate and applied principal component analysis (PCA) to these probability vectors. We found that more than 85% of variance across the dataset is explained by the first two PCs, which are themselves defined by large loadings for *Wnt*^*hi*^, *WntMapk*^*hi*^, *Mapk*^*hi*^ and high proliferative scores, highlighting a separation of cell populations across these axes (Fig. 2f and Extended Data Fig. 4e,f). There was a robust enrichment of MAPK pathway-associated genes in the AKG12D tumour epithelium when compared with APC tumours, again counteracted by MRTX1133 (Fig. 2g). Analysis of *WntMapk*^*hi*^-associated gene expression revealed enrichment of a *WntMapk*^*hi*^ population in AKG12D, which was again lost following MRTX1133 treatment. The acquisition of a *WntMapk*^*hi*^ state was broadly mirrored by patterns of enrichment for *Wnt*^*hi*^-associated gene expression (Fig. 2g). Finally, overlaying the distribution of cells from each experimental group onto the PCA demonstrated heterogeneity of cell states within genetically simple tumours, but also highlighted the impact of KRAS mutation and of subsequent KRAS inhibition on the balance of *Wnt*^*hi*^ and *WntMapk*^*hi*^ cell states (Fig. 2h). These data reinforce the existence of rapid cellular adaptive epithelial plasticity following exposure to KRAS(G12D)-targeted therapies in vivo.

## Cellular plasticity in early metastasis

**Fig. 3:**
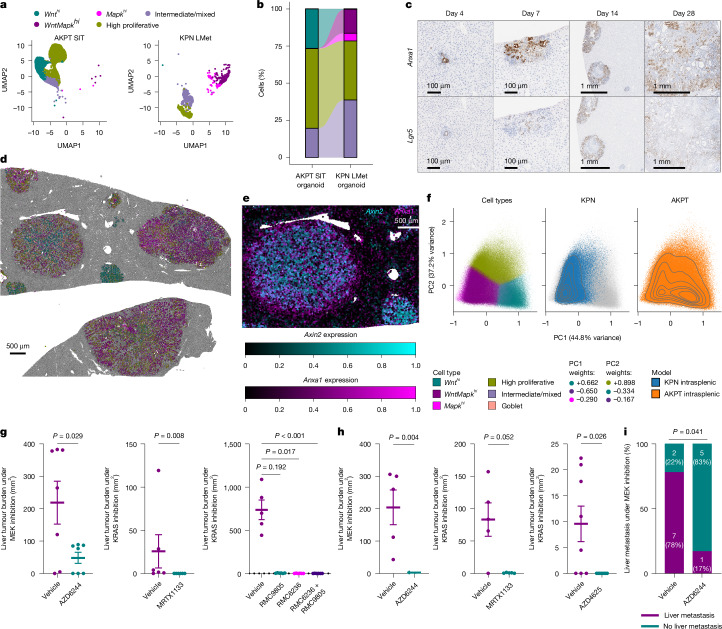
Early CRC liver metastases are *WntMapk*^*hi*^ enriched and vulnerable to MAPK inhibition. **a**, UMAP of scRNA-seq and cell-fate clusters of the tumour-derived spheroid lines AKPT small intestinal tumour (SIT) line (6,099 cells, *n* = 3) and KPN liver metastasis (LMet) line (1,501 cells, *n* = 3). **b**, Alluvial plot showing the proportion of cell fates from spheroids in panel **a**. **c**, Representative *Anxa1* and *Lgr5* in situ hybridization stains of AKPT metastases sampled 4, 7, 14 and 28 days post-intrasplenic engraftment. *n* = 3 per time point. **d**, Representative spatial profiling image of AKPT-derived liver metastasis 28 days post-intrasplenic transplantation (*n* = 3). Tumour epithelial cells are coloured by the highest probability cell-type call; non-tumour cells are in grey. **e**, Composite image of cells by scaled *Axin2* (cyan) and *Anxa1* (magenta) expression in AKPT-derived liver metastasis. **f**, PCA of cell-type probability in tumour epithelial cells. PC1 (*x* axis) describes 44.8% of variance; the top three loadings are *Wnt*^*hi*^, *WntMapk*^*hi*^ and *Mapk*^*hi*^. PC2 (*y* axis) describes 37.2% of variance; the top three loadings are high proliferative, *Wnt*^*hi*^ and *WntMapk*^*hi*^. Cells are coloured by their highest probability cell-type call, KPN intrasplenic (*n* = 4, blue) and AKPT intrasplenic (*n* = 3, orange). **g**, Liver metastatic burden in AKPT treated from 7 days post-transplantation and continued for 21 days. AZD6244 versus vehicle (*n* = 7 per group, one-tailed Mann–Whitney test); MRTX1133 versus vehicle (*n* = 6 per group, one-tailed Mann–Whitney test); and RMC9805 versus RMC6236 versus combined RMC9805–RMC6236 versus vehicle (*n* = 6 per group except *n* = 5 in vehicle, Kruskal–Wallis test) are shown. Data are mean ± s.e.m. **h**, Liver metastatic burden in KPN or KcPN treated from 7 days post-transplantation for 21 days (KPN AZD6244), 28 days (KPN MRTX1133) and 35 days (KcPN AZD4625). AZD6244 versus vehicle (*n* = 5 per group, one-tailed Mann–Whitney test); MRTX1133 versus vehicle (*n* = 5 per group, one-tailed Mann–Whitney test); and AZD4625 versus vehicle (*n* = 8 per group, two-tailed Mann–Whitney test) are shown. Data are mean ± s.e.m. **i**, Percentage of KPN with liver metastasis when treated from 85 DPI with AZD6244 (*n* = 6) or vehicle (*n* = 9; *n* (%), two-sided Fisher’s exact test).

**Extended Data Fig. 5:**
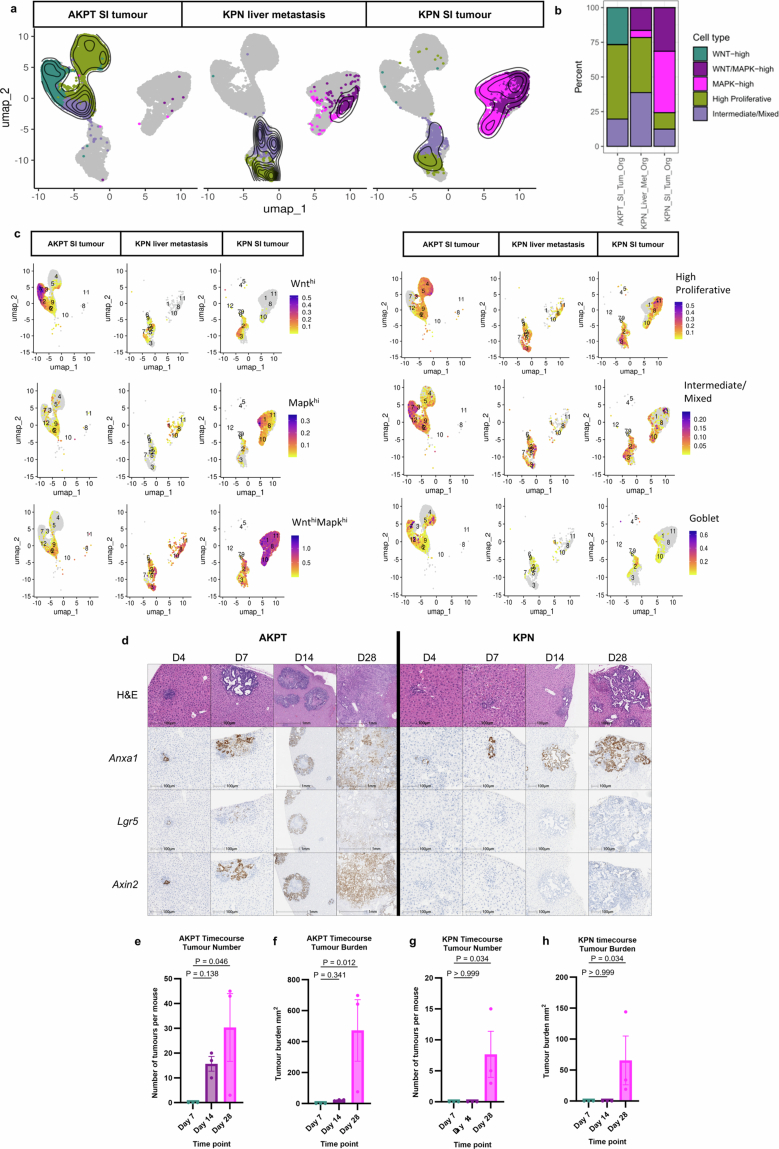
Murine tumour derived spheroids are heterogeneous and are in a Mapk^hi^ state in early liver metastasis development. A- UMAP visualisation of scRNAseq and major cell fate clusters of murine tumour derived tumour organoids. Lines are AKPT small intestinal primary tumour (AKPT SIT, 6099 cells from 3 biological replicates), KPN liver metastases (LMet) (1501 cells from 3 biological replicates) and KPN small intestinal tumour (SIT) (5251 cells from 3 biological replicates. B- Alluvial bar graph showing percentage of cells of major epithelial populations across experimental conditions form scRNAseq from Extended Data Fig. 5a. C- UMAPs of annotated gene sets in the three experimental conditions. D- Representative images of H&E and *Anxa1, Lgr5 and Axin2* ISH stains in time-course sampling of AKPT and KPN liver metastases sampled 4, 7, 14 and 28 days post intrasplenic transplantation of spheroids. n = 3 at each time point. The *Anxa1* and *Lgr5* stains of the AKPT are the same image as the main Fig. 3c. E-H- Liver tumour number and burden in AKPT and KPN intrasplenic transplantation mice across different time points. Mean ± SEM, Kruskal-Wallis test. 3 biological replicates for each time points in each model. ns= not significant *=p < 0.05.

To understand whether epithelial-state changes influenced metastatic seeding and outgrowth in KRAS-mutant CRC, we used intrasplenic orthotopic engraftment of tumour-derived spheroids, using current gold-standard models of classical (VillinCre^ERT2^ *Apc*^*fl/fl*^*Kras*^*G12D/+*^*Trp53*^*fl/fl*^*Alk5/Tgfbr1*^*fl/fl*^ (AKPT)) or serrated (VillinCre^ERT2^ *Kras*^*G12D/+*^*Trp53*^*fl/fl*^*Rosa26*^*N1icd/+*^ (KPN)) CRC^22^. Although culture conditions are known to influence the cellular fate^16^, single-cell transcriptional profiling of AKPT and KPN spheroids demonstrated substantial heterogeneity and adoption of previously described cell states (Fig. 3a,b and Extended Data Fig. 5a–c). AKPT spheroids comprised a notable subpopulation of *Wnt*^*hi*^ epithelial cells in vitro, alongside proliferative and intermediate/mixed cell types, whereas KPN spheroids were characterized by both *WntMapk*^*hi*^ and Mapk^hi^ populations. To study epithelial cell dynamics in establishing metastases, AKPT and KPN lines were engrafted via the intrasplenic route into livers of immunocompetent mice, with sampling at 4, 7, 14 or 28 days. Early hepatic lesions arising from both models exhibit robust *Anxa1* expression, with concurrent *Lgr5* and *Axin2* expression observed in early AKPT lesions (Fig. 3c and Extended Data Fig. 5d–h). Although AKPT was characterized by the *Wnt*^*hi*^ cell fate in vitro, the earliest lesions expressed high levels of *Anxa1*, a marker associated with the *WntMapk*^*hi*^ or *Mapk*^*hi*^ fates, suggesting that these states may be critical for early metastatic development in vivo. As tumours established, epithelial heterogeneity emerged in both models, with AKPT tumours exhibiting distinct regions devoid of, or replete for, *Anxa1*, *Lgr5* or *Axin2* expression, and KPN tumours exhibiting varying *Anxa1* expression, but remaining *Lgr5* and *Axin2* deficient (Fig. 3c and Extended Data Fig. 5d–h). This aligns with reports suggesting that changes in cell state are required to initiate and then maintain metastasis^12,14,15^.

**Extended Data Fig. 6:**
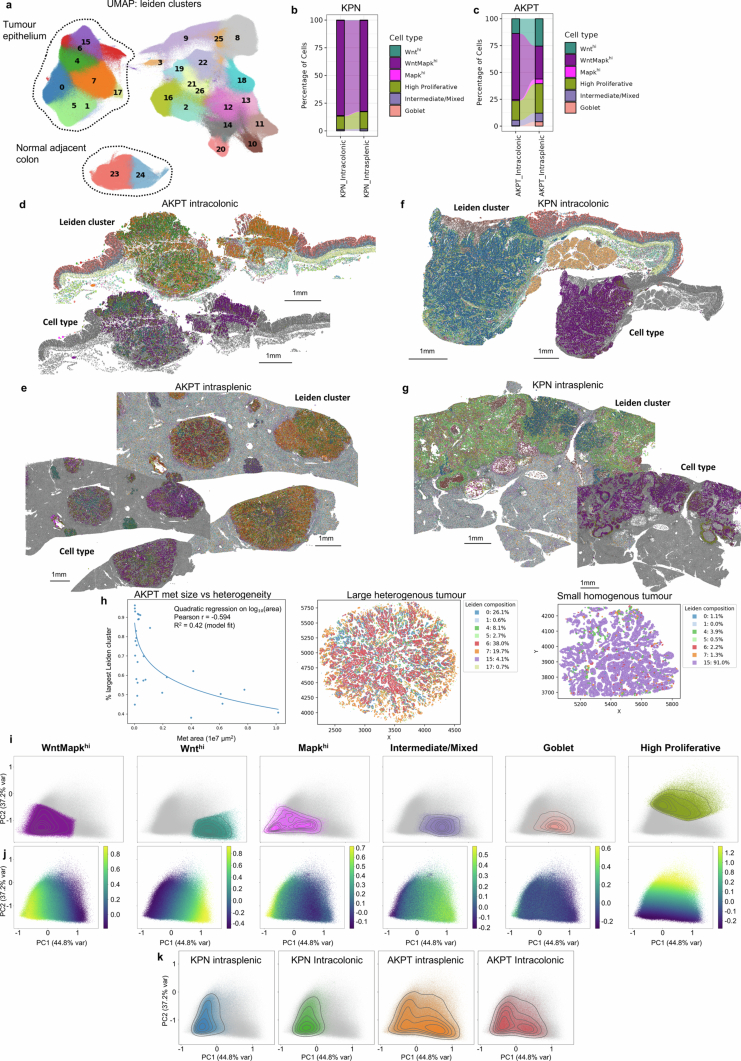
Liver metastasis are transcriptionally heterogeneous and correlates with size. A- UMAP visualisation of AKPT intracolonic, AKPT intrasplenic, KPN intracolonic, and KPN intrasplenic cells after spatial transcriptional profiling. Cell colours denote Leiden clustering. Tumour epithelial and normal adjacent colon clusters were identified based on their spatial distribution in the tissue and key markers. B- Stacked bar graph of Leiden cluster assignment of tumour epithelial cells from KPN intracolonic (n = 4) and intrasplenic (n = 4) transplanted mice. C- Stacked bar graph of Leiden cluster assignment of tumour epithelial cells from AKPT intracolonic (n = 4) and intrasplenic (n = 3) transplants. D- Representative image of spatial distribution of cells coloured by their Leiden cluster association and cell type assignment of tumour epithelial cells in AKPT spheroid derived intracolonic tumours (n = 4). Scale bar 1 mm. E- Representative image of spatial distribution of cells coloured by their Leiden cluster association and cell type assignment of tumour epithelial cells in AKPT spheroid-derived liver metastasis, 28 days post intrasplenic injection (n = 3). Scale bar 1 mm. F- Representative image of spatial distribution of cells coloured by their Leiden cluster association and cell type assignment of tumour epithelial cells in KPN organoid derived intracolonic tumours (n = 4). Scale bar 1 mm. G- Representative image of spatial distribution of cells coloured by their Leiden cluster association and cell type assignment of tumour epithelial cells in KPN organoid-derived liver metastasis, 28 days post intrasplenic injection (n = 4). Scale bar 1 mm. H- Scatter plot of individual metastasis area vs percentage largest cluster present in AKPT derived liver metastasis, pooled from three biological replicates. Representative images of large transcriptionally heterogeneous and small transcriptionally homogenous liver metastasis. Tumour epithelial cells were coloured in their respective Leiden cluster colour. I- PCA from Fig. 3f showing highest cell type call in this space. Cells are coloured by their highest probability for cell type assignment. J- Gene set scores for the individual gene sets overlayed on PCA presented in Fig. 3f. K- Cells coloured by experimental condition (AKPT intrasplenic n = 3, AKPT intracolonic n = 4, KPN intrasplenic n = 4, KPN intracolonic n = 4) overlayed on PCA presented in Fig. 3f.

We next interrogated transcriptional heterogeneity in advanced disease through spatial transcriptomic analysis of established liver metastases at 28 days post-orthotopic transplantation of AKPT and KPN spheroids alongside primary colonic tumours from the same transplanted lines. Leiden clustering was again used to broadly identify cell types, including tumour epithelial cells (Extended Data Fig. 6a). AKPT primary and metastatic tumours were heterogeneous in nature, whereas KPN tumours were mostly restricted to two clusters (Extended Data Fig. 6a,d–g). AKPT liver metastases varied in size, ranging from small epithelial clusters to large complex structures (Fig. 3d). Transcriptional heterogeneity scaled with size, as represented by the percentage abundance of the largest Leiden cluster per individual lesion, with smaller tumours more likely to exhibit transcriptional homogeneity (Extended Data Fig. 6h).

To map fates, the probability scores for each of our previously described cell fates were calculated for each tumour epithelial cell in the analysis. We found that although AKPT tumour cells adopted all described states in both intracolonic and liver metastasis, KPN tumours did not exhibit a *Wnt*^*hi*^ cell population (Extended Data Fig. 6b,c). In contrast to early lesions from time-course studies, small, homogenous AKPT metastases at 28 days comprised either *Wnt*^*hi*^ or *WntMapk*^*hi*^ populations, marked by *Axin2* expression, whereas larger lesions were heterogeneous mixtures of *Wnt*^*hi*^ and *WntMapk*^*hi*^ with distinct cellular expression of *Axin2* and *Anxa1* (Fig. 3e).

We performed PCA on probability vectors of all primary and metastatic transplant cells, once again finding that the first two principal components explained the majority (more than 80%) of variance across this dataset, and separate *Wnt*^*hi*^, *WntMapk*^*hi*^, *Mapk*^*hi*^ and highly proliferative cell populations (Fig. 3f and Extended Data Fig. 6i–k). This demonstrates the similarity between primary and metastatic lesions within genotypes, and that the *Wnt*^*hi*^ population is most prominent in the AKPT model. These data suggest that although cultured organoids have minimal *Mapk*^*hi*^ or *WntMapk*^*hi*^ populations, these are present in early seeding liver metastases, and that large, established metastases comprise heterogeneous mixtures of cell fates, including *WntMapk*^*hi*^ and *Wnt*^*hi*^.

## Early metastases respond to therapy

**Extended Data Fig. 7:**
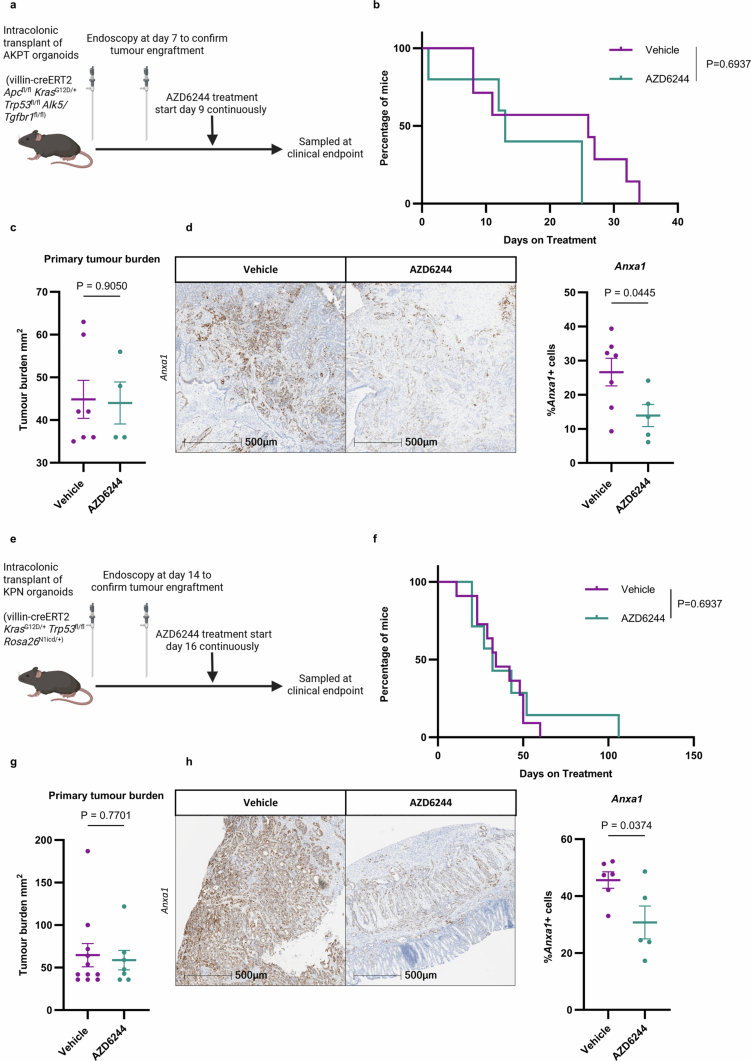
Limited impact of MEK inhibition in AKPT and KPN intracolonic transplants. A- Experimental schematic of AKPT murine derived organoid intracolonic transplantation treated with AZD6244 or vehicle control when tumour was visible on endoscope. The schematic was created in BioRender. White, M. (2025) https://BioRender.com/zz3wsxv. B- Kaplan-Meier survival curves with Log Rank test of intestinal tumour free survival of AKPT transplants treated with vehicle (n = 7) or AZD6244 (n = 5). C- Colonic tumour burden for each treatment group (vehicle, n = 7 or AZD6244, n = 4). Mean ± SEM. Two tailed t-test. D- Representative images and associated quantification of *Anxa1* ISH in vehicle (n = 7) and AZD6244 (n = 5) treated tumours. Mean ± SEM. Two tailed t-test. E- Experimental schematic of KPN murine derived organoid intracolonic transplantation treated with AZD6244 or vehicle control when tumour was visible on endoscope. The schematic was created in BioRender. White, M. (2025) https://BioRender.com/mhszqez. F- Kaplan-Meier survival curves with Log Rank test of intestinal tumour free survival of KPN transplants treated with vehicle (n = 11) or AZD6244 (n = 7). G- Colonic tumour burden each treatment group (vehicle, n = 11 or AZD6244, n = 7). Mean ± SEM. Two tailed t-test. H- Representative images and associated quantification of *Anxa1* ISH in vehicle (n = 6) and AZD6244 (n = 5) treated tumours. Mean ± SEM. Two tailed t-test.

The observation that early metastatic deposits exhibit uniformly high levels of *Anxa1*, implying transition through a single epithelial cell state (*Mapk*^*hi*^ or *WntMapk*^*hi*^) before establishment of heterogeneity, is suggestive of a window of opportunity where MAPK inhibition may be effective. We therefore tested the efficacy of MAPK-targeting agents in orthotopic metastasis models, with intervention from 7 days post-engraftment, and sampling after 21 days of treatment. In AKPT orthografts, inhibition of MEK1/2 with AZD6244 (also known as selumetinib), or inhibition of KRAS via monotherapy MRTX1133 (KRAS(G12D)), RMC9805 (KRAS(G12D-on)), RMC6236 (pan-RAS-on) or combined RMC9805–RMC6236 resulted in reduced metastasis, with almost complete response following targeted KRAS inhibition (Fig. 3g). Therapeutic intervention in the KPN model proved equally effective, with AZD6244 and MRTX1133 driving an almost complete response (Fig. 3h). Similar responses were seen in a KRAS(G12C)-mutant variant of the KPN model (VillinCre^ERT2^ *Kras*^*G12C/+*^*Trp53*^*fl/fl*^*Rosa26*^*N1icd/+*^ (KcPN)), following treatment with AZD4625 (a KRAS(G12C) inhibitor; Fig. 3h). Critically, the therapeutic sensitivity observed in early metastatic disease is not shared in established primary colonic tumours: treatment of established AKPT or KPN colonic tumours with AZD6244 had no antitumour effect, despite reducing *Anxa1* expression in both models, again reflecting an effect on epithelial identity (Extended Data Fig. 7a–h).

Although orthotopic engraftment is a powerful model of metastasis, it cannot recapitulate all aspects of the metastatic cascade. To understand the potential of MAPK targeting in a more challenging setting, we assessed early intervention with AZD6244 in the autochthonous genetically engineered mouse model (GEMM), KPN^22^. This model features robust spontaneous metastasis, representing an ideal setting to interrogate anti-metastatic therapies. Cohorts of KPN mice were administered with AZD6244 or vehicle from day 85 post-induction, in which mice exhibited intestinal tumour burden with ageing to a clinical end point. AZD6244 significantly reduced penetrance of liver metastasis (Fig. 3i). Collectively, these data demonstrate therapeutic vulnerability of early liver metastasis and may provide a clinical opportunity in the minimal residual disease or neoadjuvant setting.

## BRAF mutations favour the RSC identity

Oncogenic BRAF mutation occurs in approximately 10% of CRC tumours and is a strong activator of the MAPK pathway, and may therefore drive similar epithelial cell phenotypes as *Kras* mutation. Critically, inhibition of the MAPK pathway through combined BRAF + EGFR-targeted therapy is the established standard-of-care approach for second-line metastatic disease, although is subject to varying upfront and durations of response^2,23–25^. Given that epithelial cell-state change emerged as a feature of tumorigenesis and therapeutic resistance in *Kras*-mutant disease, we tested whether the same was true in *Braf-*mutant CRC.

**Fig. 4:**
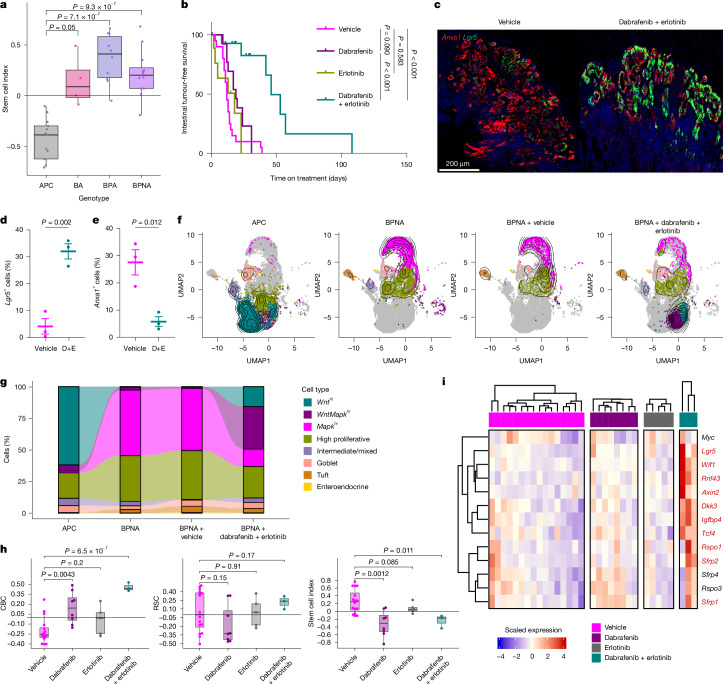
BRAF + EGFR inhibition causes adoption of a *Wnt*^*hi*^ epithelial state in *Braf*-mutant tumours. **a**, Boxplot of SCI in BRAF-driven colonic tumours compared with APC tumours. Scores are derived from RNA-seq of APC (*n* = 13) BA (*n* = 4), VillinCre^ERT2^ *Braf*^*V600E*^^/+^ *Trp53*^*fl/fl*^ *Alk5*^*fl/fl*^ (BPA, *n* = 10) and BPNA (*n* = 12) mice. Boxes are median and IQR, and whiskers extend to the minimum and maximum values reaching up to 1.5× the lower and upper IQR. Significance was determined using two-tailed Student’s *t*-tests. **b**, Kaplan–Meier survival curves with log-rank tests of intestinal tumour-free survival of BPNA mice treated with vehicle (*n* = 20), dabrafenib (*n* = 13), erlotinib (*n* = 9) or a combination of dabrafenib + erlotinib (*n* = 15) continuously from 20 DPI. Data are in days treated. **c**, Representative images of dual in situ hybridization *Anxa1* (red) and *Lgr5* (green) in BPNA tumours after 3 days of treatment with vehicle or dabrafenib + erlotinib from 27 DPI. **d**,**e**, Quantification of the percentage of *Lgr5*^*+*^ (**d**) and *Anxa*^*+*^ (**e**) cells in BPNA tumours after 3 days of treatment with vehicle or dabrafenib + erlotinib (D+E; *n* = 3 per group). Data are mean ± s.e.m. Significance was determined using a two-tailed Student’s *t*-test. **f**, UMAP of epithelial cells and cell-fate clusters with density overlay mapping from scRNA-seq of colonic tumours from APC (4,625 cells, *n* = 6 biological replicates), BPNA (3,169 cells, *n* = 5 biological replicates), BPNA mice treated with vehicle (1,305 cells, *n* = 5) and BPNA mice treated with dabrafenib + erlotinib (2,383 cells, *n* = 4). **g**, Alluvial plot showing the proportion of cell fates in panel **f**. **h**, Boxplots of single sample gene set enrichment analysis scores of CBC and RSC gene sets and SCI of RNA-seq of end point BPNA tumours in treatment groups (*n* = 16 for vehicle, *n* = 8 for dabrafenib, *n* = 5 for erlotinib and *n* = 3 for dabrafenib + erlotinib combination therapy). Boxes are median and IQR, and whiskers extend to the minimum and maximum values reaching up to 1.5× the lower and upper IQR. Significance was determined using two-tailed Student’s *t*-tests. **i**, Heatmap of scaled expression of normalized counts associated with WNT target genes in RNA-seq of BPNA colonic tumours post-treatment (for biological replicates: *n* = 16 for vehicle, *n* = 8 for dabrafenib, *n* = 5 for erlotinib and *n* = 3 for dabrafenib + erlotinib combination therapy). Statistical testing was by a two-sided Wald test; the gene symbols in red have a Benjamini–Hochberg adjusted *P* < 0.05 of vehicle versus dabrafenib + erlotinib.

**Extended Data Fig. 8:**
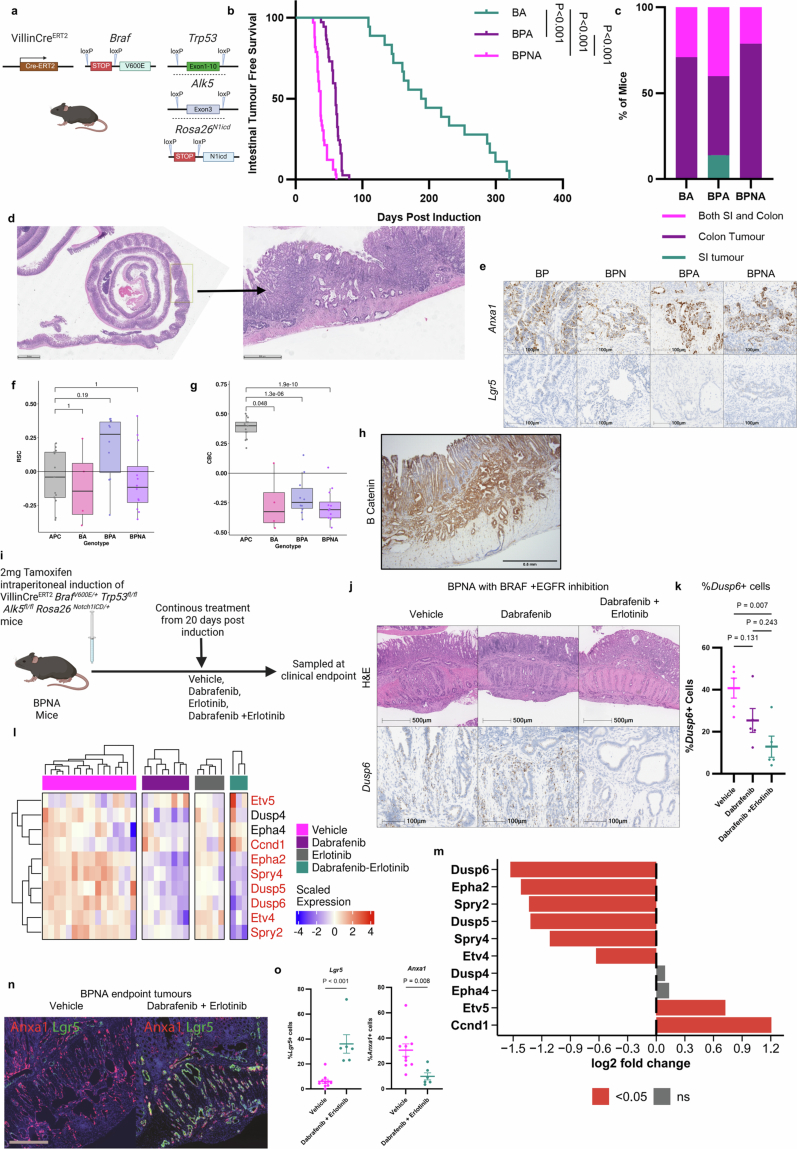
Characterisation of advanced BRAF mutant GEMMs. A- Schematic description of genetic crossing strategies to generate VillinCre^ERT2^ *Braf*^*V600E/+*^ *Trp53*^*fl/fl*^, VillinCre^ERT2^ *Braf*^*V600E/+*^ *Alk5*^*fl/f*l^ VillinCre^ERT2^ *Braf*^*V600E/+*^ *Trp53*^*fl/fl*^ *Alk5*^*fl/f*l^, VillinCre^ERT2^ *Braf*^*V600E/+*^*Trp53*^*fl/fl*^ *Rosa26*^*N1icd/+*^, and VillinCre^ERT2^ *Braf*^*−V600E*^ *Trp53*^*fl/fl*^ *Alk5*^*fl/fl*^ *Rosa26*^*N1icd/+*^ mice (BP, BA, BPA, BPN, and BPNA respectively). Cre, Cre-recombinase; ER, Oestrogen receptor; LoxP, Cre-Lox recombination site. The schematic was created in BioRender. White, M. (2025) https://BioRender.com/7yy6mmn. B- Kaplan-Meier survival curves of intestinal tumour free survival. Median tumour free survival days post induction (DPI); BA 192 days (n = 18), BPA 60 days (n = 37) and BPNA 37 days (n = 33). P-value calculated by log-rank test. C- Bar graph of primary tumour location across BA, BPA and BPNA models. D- Representative images of BPNA colon and colonic tumour from cohort (n = 33) described in (B), scale bar 2 mm low power image, 500μm high power image. E- Representative images of *Anxa1* and *Lgr5* ISH in tumours of each genotype (n = 5 each genotype). Scale bar 100μm. F-G- Boxplots of single sample gene set enrichment analysis (ssGSEA) scores of crypt base columnar (CBC) and regenerative stem cell (RSC) gene sets from bulk RNA sequencing of VillinCre^ERT2^ *Apc*^fl/fl^ (APC, n = 13), BA (n = 4), BPA (n = 10), BPNA (n = 12) mice. Boxes are median and IQR, whiskers extend to minimum and maximum values reaching up to 1.5*lower and upper IQR. Two-tailed t-tests. H- Representative images of a BPNA colonic tumour stained with β-Catenin IHC staining showing membranous rather than nuclear staining. n = 3. Scale bar 500μm. I- Experimental schematic of VillinCre^ERT2^ *Braf*^*−V600E*^ *Trp53*^*fl/fl*^ *Alk5*^*fl/fl*^ *Rosa26*^*N1icd/+*^ (BPNA) mice treated from 20 days post induction. The schematic was created in BioRender. White, M. (2025) https://BioRender.com/a9j6uli. J- Representative images of H&E and *Dusp6* ISH in BPNA colonic tumours under each treatment condition. K- Quantification of *Dusp6* positive cells in tumour area across each group (n = 5 in each group). Mean ± SEM, One-way ANOVA with Tukey correction for multiple comparisons L- Heatmap of scaled expression of normalised counts associated with MAPK activation markers identified by bulk RNAseq in each treatment condition of BPNA tumours (vehicle n = 16, dabrafenib n = 8, erlotinib n = 5, dabrafenib + erlotinib combination therapy n = 3). Statistical testing by two-sided Wald test, red gene have a Benjamini-Hochberg adjusted p-value < 0.05 for dabrafenib + erlotinib vs vehicle. M- Log2 fold change of MAPK activation target genes from bulk RNA sequencing of BPNA tumours treated with dabrafenib + erlotinib vs vehicle control. Statistical testing by two-sided Wald test, red bars have a Benjamini-Hochberg adjusted p-value < 0.05. ns= not significant. N- Representative images of dual FISH *Anxa1* (red) and *Lgr5* (green) in endpoint BPNA tumours after prolonged treatment from Day 20 post induction until clinical endpoint with vehicle (n = 10) or dabrafenib and erlotinib (n = 6). Scale bar 250μm O- Associated quantification of *Lgr5+* and *Anxa1+* cells in endpoint BPNA tumours after prolonged treatment from Day 20 post induction ongoing until clinical endpoint with vehicle (n = 10) or dabrafenib and erlotinib (n = 6). Mean ± SEM two-tailed t-test.

We have previously described a robust genetically engineered model of right-sided CRC, driven by oncogenic BRAF mutation and *TGFBR1*/*Alk5* deletion (VillinCre^ERT2^ *Braf*^*V600E/+*^*Alk5*^*fl/fl*^ (BA))^26^. This was augmented through deletion of *Trp53* and/or transgenic activation of the Notch pathway, both common features of human BRAF-mutant CRC, and drivers of aggressive tumour features in vivo^1,22,27,28^. The resultant model, VillinCre^ERT2^ *Braf*^*V600E/+*^*Trp53*^*fl/fl*^*Rosa26*^*N1icd/+*^*Alk5*^*fl/fl*^ (BPNA; Extended Data Fig. 8a), rapidly developed invasive right-sided colonic tumours and recapitulated many features of human BRAF-mutant disease (Extended Data Fig. 8b–d). Strong *Anxa1* expression and an absence of *Lgr5* expression were observed universally in BRAF-driven tumours (Extended Data Fig. 8e), with evident suppression of the CBC signature, enrichment of RSC signature and an SCI value that contrasted with APC-deficient tumours (Fig. 4a and Extended Data Fig. 8f,g). In line with the absence of WNT-associated features, BPNA tumours lacked nuclear accumulation of β-catenin, indicative of inactive WNT signalling (Extended Data Fig. 8h). Given the rapid, robust and reproducible tumour development in the BPNA and strong alignment to human disease, it represents a powerful platform for preclinical therapeutic testing.

## BRAF therapies alter cellular identity

Although MAPK targeting in the AKG12D model enriched for the *Wnt*^*hi*^ epithelial population, this occurred in the context of *Apc* mutation. The BPNA model lacks WNT pathway mutation or WNT activation, and human *BRAF*-mutant CRCs rarely feature *APC* mutation, implying that in response to therapeutic pressure, these tumours may be unable to rapidly adopt an alternative WNT-enriched fate. To test this hypothesis and the impact of standard-of-care BRAF + EGFR inhibition, we challenged BPNA mice with dabrafenib (a BRAF inhibitor) and erlotinib (an EGFR inhibitor) either as monotherapy or in combination (Extended Data Fig. 8i). Although these agents were ineffective as monotherapy, as observed clinically^29^, combined administration doubled overall tumour-free survival (Fig. 4b). Despite increased survival, BPNA mice ultimately developed colonic tumours, suggestive of therapeutic resistance. These putatively resistant tumours retained invasive properties, but appeared to exhibit reduced epithelial MAPK activity, marked by reduced expression of *Dusp6* and multiple other MAPK target genes (Extended Data Fig. 8j–l). This implies that MAPK reactivation is not the key driver of resistance in this model, and that epithelial cell plasticity may have a role.

**Extended Data Fig. 9:**
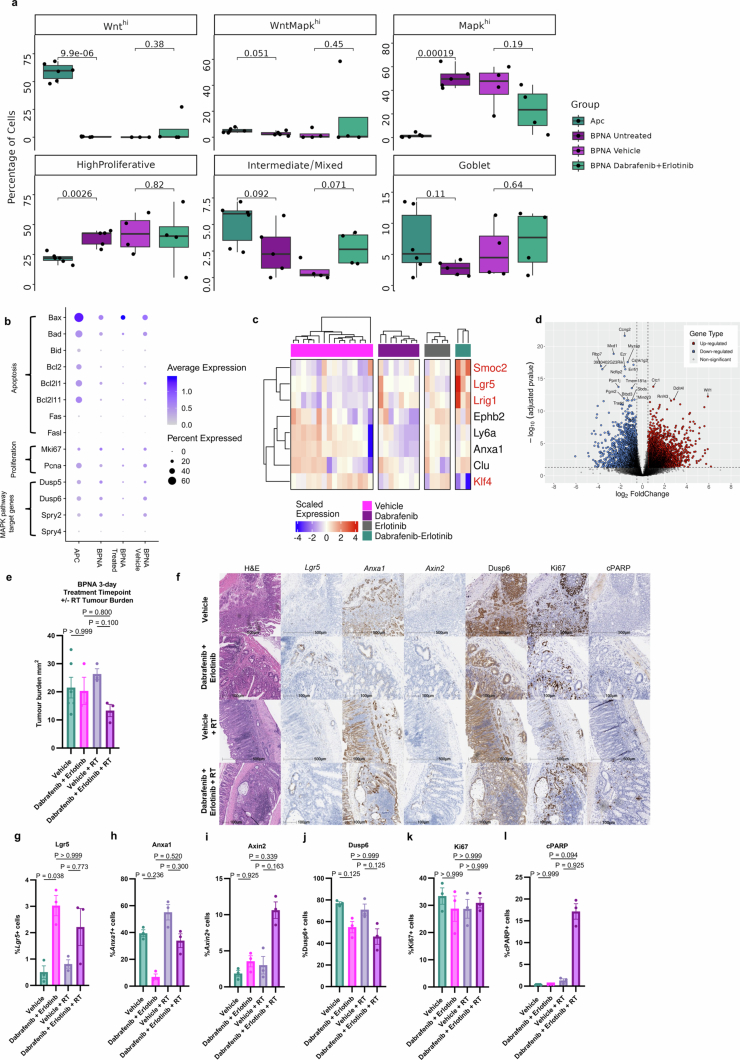
Epithelial cellular plasticity in response to BRAF+EGFR inhibition in BPNA tumours and induces apoptosis with Lgr5 targeting radiotherapy. A- Boxplots comparing the cell type proportion of four experimental conditions. Each dot represents the cell type proportion of each sample. Colours indicated each experimental condition. Boxes are median and IQR, whiskers extend to minimum and maximum values reaching up to 1.5*lower and upper IQR. Two-tailed t-test. B- Individual marker genes relating to apoptosis, proliferation and MAPK-pathway signalling across each experimental condition from sc RNAseq. Dot size represents percentage of expressing cells and colour indicates average expression. C- Heatmap of scaled expression of normalised counts of genes associated with the CBC or RSC phenotype by bulk RNAseq of intracolonic tumours from BPNA tumours in annotated treatment conditions (vehicle n = 16, dabrafenib n = 8, erlotinib n = 5, dabrafenib + erlotinib combination therapy n = 3). Statistical testing by two-sided Wald test, red gene symbols have a Benjamini-Hochberg adjusted p-value < 0.05. D- Volcano plot of showing up and down regulated genes post dabrafenib + erlotinib compared to vehicle controls in BPNA mice. Statistical testing by two-sided Wald test. E- Tumour burden (mm^2^) in VillinCre^ERT2^ *Braf*^*−V600E*^ *Trp53*^*fl/fl*^ *Alk5*^*fl/fl*^ *Rosa26*^*N1icd/+*^ (BPNA) mice following 3-days of vehicle (n = 6) or 3-days of dabrafenib + erlotinib (n = 3) combination treatment from day 27 post-induction; or 3-days of vehicle + a single dose of Radiotherapy (RT) (4 Gy) (n = 3) or 3-days of dabrafenib + erlotinib (n = 3) combination treatment + a single dose of RT (4 Gy). Mean ± SEM. Kruskal-Wallis test with Dunn’s correction for multiple comparisons. F- Representative images of H&E, *Lgr5, Anxa1* and *Axin2* ISH, p-ERK, Ki67 and c-PARP IHC in BPNA mice following 3-days of vehicle (n = 3), dabrafenib + erlotinib (n = 3), vehicle + RT (n = 3), dabrafenib + erlotinib + RT (n = 3) from 27 DPI. Scale bars 500μm and 100μm as indicated. G-L- Quantification of indicated ISH (*Lgr5, Anxa1* and *Axin2*) and IHC (Dusp6, Ki67 and c-PARP) staining in BPNA mice following 3-days of vehicle (n = 3), dabrafenib + erlotinib (n = 3), vehicle + RT (n = 3), dabrafenib + erlotinib + RT (n = 3) from 27 DPI. Mean ± SEM. Kruskal-Wallis test with Dunn’s correction for multiple comparisons.

After only 3 days of treatment, BPNA tumours displayed a clear switch from an *Anxa1*^*hi*^, *Lgr5*^*low*^ tumour epithelium to an *Anxa1*^*low*^, *Lgr5*^*hi*^ tumour epithelium, a pattern sustained in chronically treated, resistant end point tumours (Fig. 4c–e and Extended Data Fig. 8m–o). Single-cell transcriptomic analysis of APC and BPNA tumours, as well as end point BPNA tumours treated with vehicle or BRAF + EGFR inhibition highlighted substantial epithelial plasticity (Fig. 4f,g and Extended Data Fig. 9a). BPNA tumours were characterized by a large *Mapk*^*hi*^ population, and paucity of *Wnt*^*hi*^ cells, contrasting APC tumours. Treatment of BPNA tumours with BRAF + EGFR inhibitors resulted in marked reduction of the *Mapk*^*hi*^ population and expansion of *Wnt*^*hi*^ and *WntMapk*^*hi*^ populations. This enrichment of *Wnt*^*hi*^ and *WntMapk*^*hi*^ populations occurred alongside suppression of MAPK target genes, and in the absence of changes in other populations, induction of apoptosis or reduction of proliferation markers (Extended Data Fig. 9b). This suggests that despite the lack of WNT pathway mutations, BPNA tumour epithelium can shift phenotype in response to targeted therapy to maintain tumour cell viability, growth and progression.

Bulk RNA-seq of post-treatment, resistant tumours recapitulated many scRNA-seq findings. *Lgr5* and *Smoc2* were induced following treatment, alongside enrichment of the CBC signature, and consequent effect on SCI, albeit without effect on RSC (Fig. 4h and Extended Data Fig. 9c). *Wif1* and *Rnf43* (WNT-negative regulators and markers of WNT activation) were among the most upregulated genes post-treatment, whereas broader examination demonstrating robust WNT pathway activation in response to treatment (Fig. 4i and Extended Data Fig. 9d).

Given that MAPK inhibition drives tumour cells towards an *Lgr5*-enriched state and the *Lgr5*^*+*^ intestinal stem cell population exhibits sensitivity to DNA damage^30,31^, we reasoned that BRAF + EGFR inhibition might sensitize BPNA tumours to radiotherapy. BPNA mice were treated with BRAF + EGFR inhibitors from day 27 post-induction for 3 days, followed by whole-body irradiation (4 Gy), with samples taken 6 h later. This resulted in an increased abundance of *Lgr5*^*+*^ cells, and critically a marked induction of pro-apoptotic signalling, marked by cPARP (Extended Data Fig. 9e–l), indicating improved therapeutic impact through manipulation of epithelial cell fate. These data demonstrate that BRAF + EGFR inhibition causes *Braf*-mutant tumours to shift from a MAPK-driven state towards WNT activation, and that this adaptive response represents a key therapeutic resistance mechanism akin to that described for *Kras*-mutant disease.

## RNF43 loss restricts cellular plasticity

**Fig. 5:**
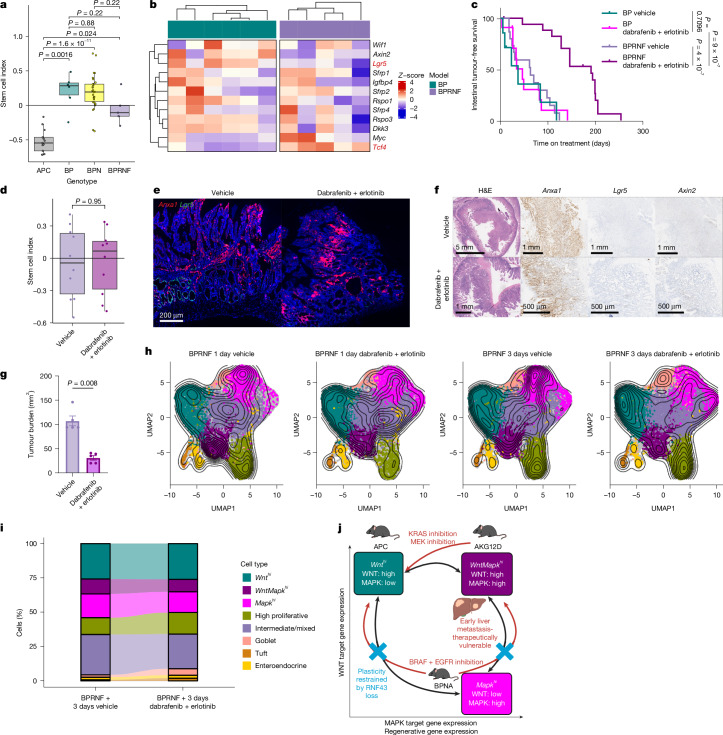
RNF43 loss sensitizes to BRAF + EGFR inhibition via restricting tumour epithelial plasticity. **a**, Boxplot of SCI in APC or BRAF-driven small intestinal tumours. Scores are from RNA-seq of APC (*n *= 13), BP (*n* = 6), BPN (*n* = 30) and BPRNF (*n* = 5) mice. The boxes are median and IQR, and whiskers extend to the minimum and maximum values reaching up to 1.5× the lower and upper IQR. Significance was determined using two-tailed Student’s *t*-tests. **b**, Heatmap of scaled expression of normalized counts associated with WNT target genes from RNA-seq of BP (*n* = 6) and BPRNF (*n* = 5) tumours. The gene symbols in red have a Benjamini–Hochberg adjusted *P* < 0.05. **c**, Kaplan–Meier curves with log-rank tests of intestinal tumour-free survival of BP mice treated with vehicle (*n* = 7) or dabrafenib + erlotinib (*n* = 11) and BPRNF mice treated with vehicle (*n* = 15) or dabrafenib + erlotinib (*n* = 18) continuously from 130 DPI. Plotted are days treated. **d**, Boxplot of SCI of end point BPRNF small intestinal tumours treated with vehicle (*n* = 10) or dabrafenib + erlotinib (*n* = 10). The boxes are median and IQR, and the whiskers extend to the minimum and maximum values reaching up to 1.5× the lower and upper IQR. Significance was determined using a two-tailed Student’s *t*-test. **e**, Representative dual in situ hybridization* Anxa1* (red) and *Lgr5* (green) images in BPRNF end point tumours treated with vehicle (*n* = 5) or dabrafenib + erlotinib (*n* = 2). **f**, Representative haematoxylin and eosin (H&E), *Anxa1*, *Lgr5* and *Axin2* in situ hybridization images in BPRNF tumours following 3 days of vehicle (*n* = 5) or dabrafenib + erlotinib (*n* = 5) from 130 DPI. **g**, Primary tumour burden in BPRNF mice following 3 days of vehicle (*n* = 5) or dabrafenib + erlotinib (*n* = 5) treatment from 130 DPI. Data are mean ± s.e.m. Significance was determined using a two-tailed Mann–Whitney test. **h**, UMAP of epithelial cells and cell-fate clusters with density overlay mapping from scRNA-seq of BPRNF small intestinal tumours treated with vehicle (8,869 cells, *n* = 3) and dabrafenib + erlotinib (11,379 cells, *n* = 4) for 1 day or vehicle (20,957 cells, *n* = 4) and dabrafenib + erlotinib (13,140 cells, *n* = 5) for 3 days. **i**, Alluvial bar graph showing the proportion of cell states between BPRNF mice treated with vehicle or the dabrafenib + erlotinib combination for 3 days in panel **h**. **j**, Graphical summary representing the key epithelial cell states in CRC and response to therapy. The *x* axis represents increasing expression of MAPK and regenerative-associated genes, and the *y* axis represents expression of WNT pathway targets. The red arrows indicate the ability of GEMMs to shift epithelial cell state under treatment pressure. The schematic was created in BioRender. White, M. (2025) https://BioRender.com/z7mw8xa.

**Extended Data Fig. 10:**
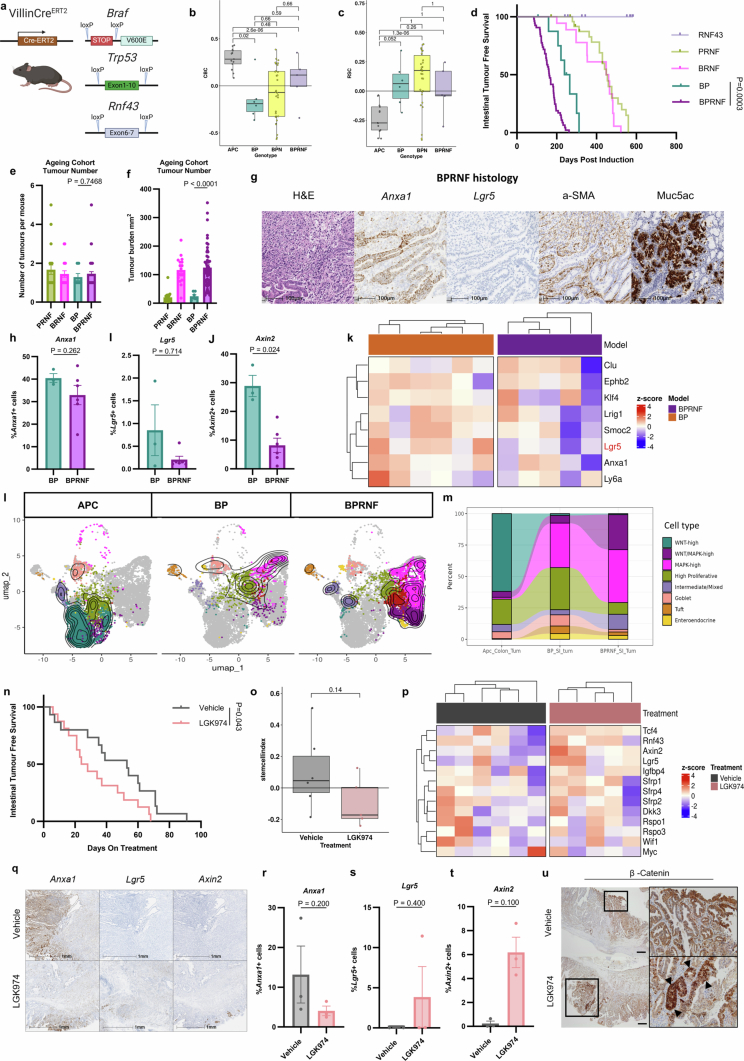
RNF43 does not drive a Wnt active phenotype in the context of Braf mutation and unaffected by Wnt ligand reduction. A- Schematic description of genetic crossing strategies to generate VillinCre^ERT2^ *Rnf43*^fl/fl^ (RNF43), VillinCre^ERT2^ *Braf*^*V600E/+*^ *Trp53*^*fl/fl*^ (BP), VillinCre^ERT2^ *Trp53*^*fl/fl*^ *Rnf43*^*fl/fl*^ (PRNF), VillinCre^ERT2^ *Braf*^*V600E/+*^ *Rnf43*^*fl/f*^ (BRNF), VillinCre^ERT2^ *Braf*^*V600E/+*^ *Trp53*^*fl/fl*^ *Rnf43*^*fl/fl*^ mice (BPRNF). Cre, Cre-recombinase; ER, Oestrogen receptor; LoxP, Cre-Lox recombination site. The schematic was created in BioRender. White, M. (2025) https://BioRender.com/k5ttyup. B-C- Boxplots of single sample gene set enrichment analysis (ssGSEA) scores of crypt base columnar (CBC) and regenerative stem cell (RSC) gene sets from bulk RNA sequencing of endpoint Braf-driven small intestinal tumours (APC, n = 13, BP, n = 6, BPN, n = 30, BPRNF, n = 5). Boxes are median and IQR, whiskers extend to minimum and maximum values reaching up to 1.5*lower and upper IQR. Two-tailed t-test. D- Kaplan-Meier survival curves with Log Rank test of intestinal tumour free survival of RNF43 (n = 13), PRNF (n = 26), BRNF (n = 18), BP (n = 9) and BPRNF (n = 55) mice aged until clinical endpoint. Data presented as days post induction (DPI). Median tumour free survival: RNF43 undefined, PRNF 449 days, BRNF 454 days, BP 252 days, BPRNF 162 days. E-F- Tumour number and tumour burden (mm^2^) in PRNF (n = 21), BRNF (n = 18), BP (n = 7) and BPRNF (n = 52) mice aged until clinical endpoint. Mean ± SEM. Kruskal-Wallis test with Dunn’s correction for multiple comparisons. Total mouse number (n) for tumour number (E) and tumour burden (F) is lower as mice sampled for reasons other than intestinal tumorigenesis have been removed. G- Representative image of H&E, *Anxa1* and *Lgr5* ISH, a-SMA and Muc5ac IHC staining in BPRNF intestinal tumours at clinical endpoint. Scale 100μm. H-J- Quantification of indicated ISH (*Anxa1, Lgr5* and *Axin2*) in BP (n = 3) and BPRNF (n = 6) intestinal tumours at clinical endpoint. Mean ± SEM. Two-tailed Mann-Whitney. K- Heatmap of scaled expression of normalised counts associated with key RSC and CBC genes identified by bulkRNAseq of tumours from BP (n = 6) and BPRNF (n = 5) mice. Statistical testing by two-sided Wald test, gene symbols in red have a Benjamini-Hochberg adjusted p-value < 0.05. L- UMAP visualisation of epithelial cells and major cell fate clusters with density overlay mapping from scRNAseq of colonic tumours from APC (4625 cells from 6 biological replicates), BP (1616 cells from 6 biological replicates) and BPRNF (5676 cells from 8 biological replicates) mice. M- Alluvial bar graph showing percentage of cells of major epithelial populations across experimental conditions from scRNAseq from Extended Data Fig. 10l. N- Kaplan-Meier survival curves with Log Rank tests of intestinal tumour free survival of BPRNF mice treated continuously with vehicle (n = 15) or LGK974 (PORCN inhibitor, n = 16) from 130 DPI. Data presented as days on treatment. O- Boxplot of single sample gene set enrichment analysis (ssGSEA) scores of stem cell index (SCI) from bulk RNA sequence (bulk RNAseq) of BPRNF tumours treated with either vehicle (n = 6) or LGK974 (n = 5). Boxes are median and IQR, whiskers extend to minimum and maximum values reaching up to 1.5*lower and upper IQR. Two-tailed t-test. P- Heatmap of scaled expression of normalised counts associated with Wnt target genes as identified by bulk RNAseq of tumours from BPRNF mice treated with vehicle (n = 6) or LGK974 (n = 5). None of the displayed genes have a Benjamini-Hochberg adjusted p-value < 0.05. Q- Representative images of *Anxa1, Lgr5* and *Axin2* ISH in BPRNF mice treated with vehicle (n = 3) or LGK974 (n = 3) from 130 DPI. Scale 1 mm. R-T- Quantification of indicated ISH (*Anxa1, Lgr5* and *Axin2*) in BPRNF mice treated with vehicle (n = 3) or LGK974 (n = 3). Mean ± SEM. Two-tailed Mann-Whitney. U- Representative images of β*-*Catenin staining in BPRNF mice treated with vehicle (n = 3) or LGK974 (n = 3). Black arrows indicate clones of β*-*Catenin positivity. Scale 500μm.

Upon acquisition of resistance to BRAF + EGFR-targeted therapies in BPNA tumours, *Rnf43* was significantly upregulated (Fig. 4i). Loss-of-function mutation of *RNF43* frequently occurs in human *BRAF*-mutant CRC and predicts exceptional responses to BRAF + EGFR inhibition^1,23,32–35^. Therefore, we explored in vivo loss of RNF43 in *Braf*-mutant disease by generating VillinCre^ERT2^ *Braf*^*V600E/+*^*Trp53*^*fl/fl*^*Rnf43*^*fl/fl*^ (BPRNF) mice (Extended Data Fig. 10a). This resulted in development of a small number of highly invasive, stromal-rich intestinal tumours. Transcriptionally, these resembled VillinCre^ERT2^ *Braf*^*V600E/+*^*Trp53*^*fl/fl*^ (BP) and strongly contrasted APC tumours with respect to SCI (Fig. 5a and Extended Data Fig. 10b–g). Despite a WNT pathway-activating mutation, primary BPRNF tumours broadly aligned to BP tumours with respect to WNT target gene expression, and were characterized by extensive expression of *Anxa1* and the absence of prominent *Lgr5* expression (Fig. 5b and Extended Data Fig. 10h–k). This skew from a WNT-driven epithelium in BPRNF and BP tumours was confirmed at the single-cell level, with both BPRNF and BP tumours largely devoid of *Wnt*^*hi*^ cells, contrasting markedly to APC tumours (Extended Data Fig. 10l,m). Both BP and BPRNF tumours exhibited enrichment of a *Mapk*^*hi*^ population, with BPRNF tumours also characterized by a substantial *WntMapk*^*hi*^ population. This demonstrates that although bulk transcriptomics and histological staining of stem markers indicate similarities between BP and BPRNF tumours, the increased granularity of scRNA-seq uncovers a shift towards a *WntMapk*^*hi*^ state in BPRNF.

The increased *WntMapk*^*hi*^ population in BPRNF compared with BP may be a consequence of ligand-dependent WNT activation. We therefore therapeutically targeted porcupine (PORCN), an *O*-acyltransferase required for efficient secretion of functional WNT ligands, inhibition of which has some clinical activity in late-stage, RNF43-mutant CRC^36^. We treated BPRNF mice with LGK974 (a PORCN inhibitor) continuously from 130 DPI up to clinical end point. This resulted in a slight, yet significant acceleration of disease, perhaps attributable to the effect of LGK974 on normal intestinal homeostasis^37^ (Extended Data Fig. 10n). Bulk transcriptional analysis of LGK974-treated tumours suggested no effect on CBC, RSC or SCI index, and no effect on WNT target gene expression, whereas stem cell marker expression detected by in situ hybridization was also unaffected (Extended Data Fig. 10o–t). Although nuclear β-catenin was broadly undetectable in BPRNF tumours, distinct areas exhibiting both nuclear accumulation of β-catenin and elevated expression of the WNT target gene *Axin2* were detected following LGK974 treatment, suggesting clonal outgrowth of ligand-independent WNT pathway-mutant cells, which in turn may also underpin accelerated disease (Extended Data Fig. 10u).

**Extended Data Fig. 11:**
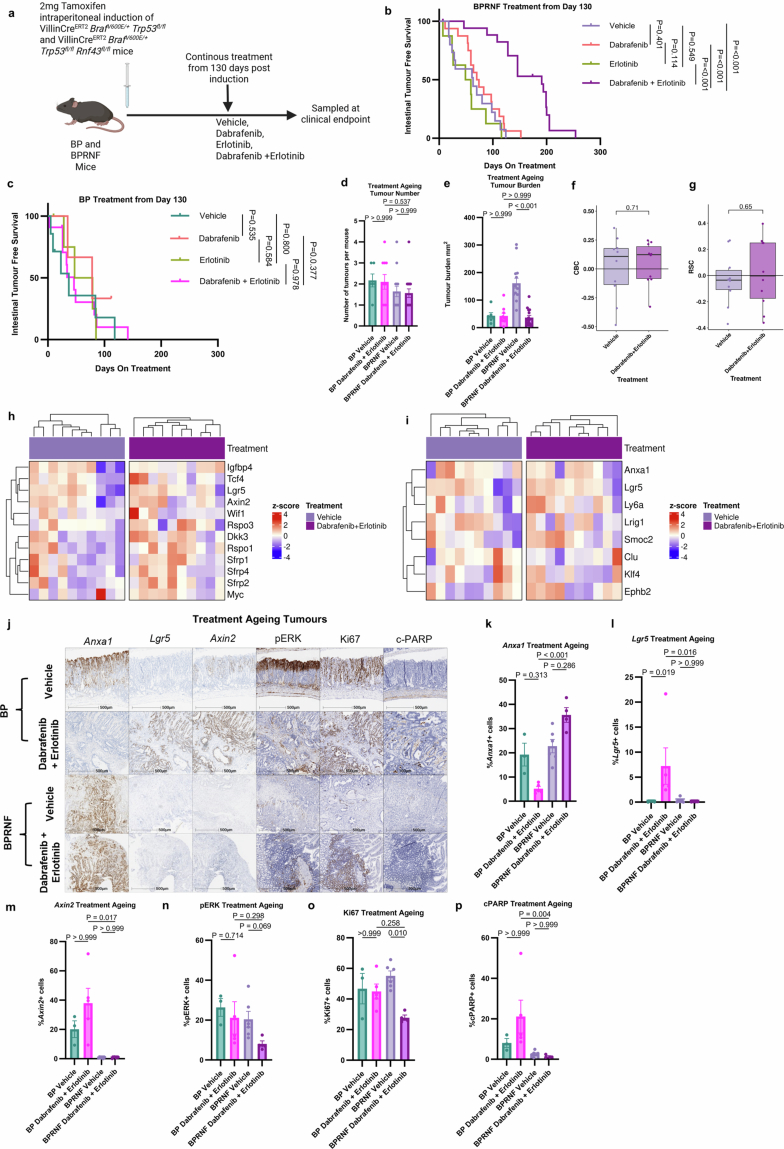
RNF43 loss prevents cell state plasticity resistance in Braf mutant tumours. A- Experimental schematic of BP and BPRNF mice treated from 130 DPI. The schematic was created in BioRender. White, M. (2025) https://BioRender.com/effw414. B- Kaplan-Meier survival curves with Log Rank tests of intestinal tumour free survival of BPRNF mice treated continuously with vehicle (n = 15), dabrafenib (n = 16), erlotinib (n = 8) or dabrafenib + erlotinib (n = 18) combination from 130 DPI. Data presented as days on treatment. Median tumour free survival: vehicle 63 days, dabrafenib 72 days, erlotinib 54 days, dabrafenib + erlotinib 191 days. C- Kaplan-Meier survival curves with Log Rank tests of intestinal tumour free survival of BP mice treated continuously with vehicle (n = 7), dabrafenib (n = 3), erlotinib (n = 5) or dabrafenib + erlotinib (n = 11) combination from 130 DPI. Data presented as days on treatment. Median tumour free survival: vehicle 37 days, dabrafenib 78 days, erlotinib 63 days, dabrafenib + erlotinib 46 days. D-E- Tumour number and tumour burden (mm^2^) in BP vehicle (n = 6), BP dabrafenib + erlotinib (n = 10), BPRNF vehicle (n = 14), BPRNF dabrafenib + erlotinib (n = 16) mice. Mean ± SEM. Kruskal-Wallis test with Dunn’s correction for multiple comparisons. Total mouse number (n) for tumour number (D) and tumour burden (E) is lower as mice sampled for reasons other than intestinal tumorigenesis have been removed. F-G- Boxplot of bulk crypt base columnar (CBC) and regenerative stem cell (RSC) score of BPRNF mice treated with either vehicle or dabrafenib + erlotinib (n = 10 both conditions). Boxes are median and IQR, whiskers extend to minimum and maximum values reaching up to 1.5*lower and upper IQR. Two-tailed t-test. H-I- Heatmap of scaled expression of normalised counts associated with Wnt target genes and stem cell genes respectively as identified by bulkRNAseq of tumours from BPRNF mice treated with vehicle (n = 10) or dabrafenib + erlotinib (n = 10). Statistical testing by two-sided Wald test, no displayed genes have a Benjamini-Hochberg adjusted p-value < 0.05. J- Representative images of *Anxa1, Lgr5* and *Axin2* ISH, p-ERK, Ki67 and c-PARP IHC in BP vehicle (n = 3), BP dabrafenib + erlotinib (n = 5), BPRNF vehicle (n = 6) and BPRNF dabrafenib + erlotinib (n = 4) mice treated continuously from 130 DPI. Scale 500μm as indicated. K-P- Associated quantification of indicated ISH (*Anxa1, Lgr5* and *Axin2*) and IHC (p-ERK, Ki67 and c-PARP) in BP vehicle (n = 3), BP dabrafenib + erlotinib (n = 5), BPRNF vehicle (n = 6) and BPRNF dabrafenib + erlotinib (n = 4) mice treated continuously from 130 DPI. Mean ± SEM. Kruskal-Wallis test with Dunn’s correction for multiple comparisons.

The acquisition of a *WntMapk*^*hi*^ state at the expense of *Mapk*^*hi*^, under both therapeutic pressure in the BPNA model, and as a result of *Rnf43* loss in the BP model poses an intriguing question of whether a BRAF-driven tumour that has adopted a basal *WntMapk*^*hi*^ state can respond to therapeutic challenge through further epithelial state changes, and, if not, does this affect therapeutic efficacy. This is pertinent given clinical responses to BRAF + EGFR inhibition in RNF43-mutant cancers. Tumour-bearing BP and BPRNF mice were subject to BRAF + EGFR inhibition from 130 DPI to clinical end point (Extended Data Fig. 11a), resulting in dramatic slowing of disease in BPRNF mice with limited effect in BP mice (Fig. 5c and Extended Data Fig. 11b–e). Contrasting the BPNA model, bulk RNA-seq of post-treatment BPRNF tumours showed no evidence of alteration in epithelial cell fate and no effect on SCI, RSC or CBC (Fig. 5d and Extended Data Fig. 11f,g). Consistently, there were no significant changes in WNT target gene expression, nor any induction of the stem cell markers *Lgr5* or *Smoc2* (Extended Data Fig. 11h,i). This is contrasted by tumours from BP mice, which did not respond to therapy, in which an increased abundance of *Lgr5* and *Axin2* and decreased *Anxa1* expression were observed (Fig. 5e and Extended Data Fig. 11j–p).

**Extended Data Fig. 12:**
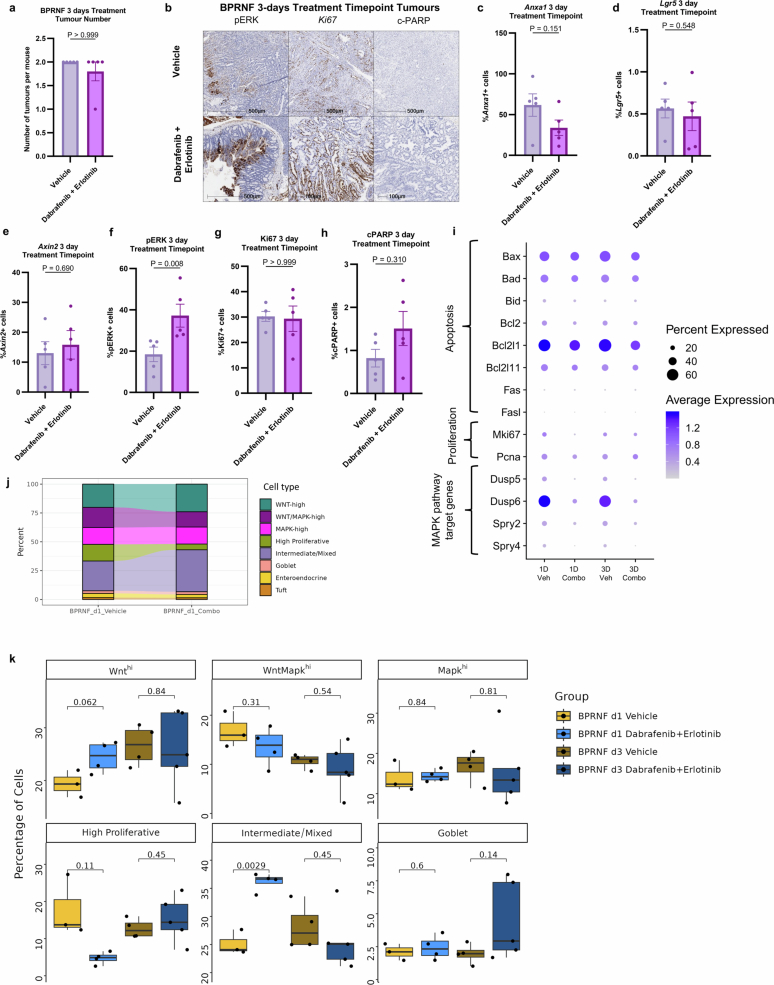
Short treatment exposure does not impact BPRNF epithelial phenotype and RNF43 loss prevents epithelial cellular plasticity in response to BRAF+EGFR inhibition. A- Tumour number in VillinCre^ERT2^ *Braf*^*V600E/+*^ *Trp53*^*fl/fl*^ *Rnf43*^*fl/fl*^ (BPRNF) mice treated with vehicle (n = 5) or dabrafenib + erlotinib (n = 5) for 3 days from 130 DPI. Mean ± SEM. Two-tailed Mann-Whitney. B- Representative images of p-ERK, Ki67 and c-PARP IHC in BPRNF mice treated with vehicle (n = 5) or dabrafenib + erlotinib (n = 5) for 3 days from day 130 post-induction. Scale bars 500μm and 100μm as indicated. C-H- Associated quantification of indicated ISH (Anxa1, Lgr5 and Axin2) and IHC (p-ERK, Ki67 and c-PARP) in BPRNF mice treated with vehicle (n = 5) or dabrafenib + erlotinib (n = 5) for 3 days from day 130 post-induction. Mean ± SEM. Two-tailed Mann-Whitney test. I- Dot plot showing the expression of genes associated with apoptosis, proliferation and MAPK pathway targets between experimental conditions in BPRNF mice treated with vehicle or dabrafenib + erlotinib combination for 1 day and 3 days from scRNAseq. J- Alluvial bar graph showing percentage of cells of major epithelial populations between experimental conditions in BPRNF mice treated with vehicle or dabrafenib + erlotinib combination for 1 day from scRNAseq from Fig. 5h. K- Boxplots comparing the cell type proportion of four experimental conditions. Each dot represents the cell type proportion of each sample. Colours indicated each experimental condition. Boxes are median and IQR, whiskers extend to minimum and maximum values reaching up to 1.5*lower and upper IQR. Two-tailed t-test.

Given the exceptional response in BPRNF mice and chronic treatment pressure, the lack of epithelial cell-state change may result from emergent resistant clonal outgrowth driven by acquired mutations that support the *WntMapk*^*hi*^ and *Mapk*^*hi*^ states. Therefore, we examined the acute impact of BRAF + EGFR inhibition in tumour-bearing BPRNF mice, over a timescale (3 days) in which epithelial-state changes were observed in BPNA and KRAS(G12D) models (Extended Data Fig. 12a). Here tumour burden was drastically reduced following BRAF + EGFR treatment, driven predominantly by robust tumour stromal regression (Fig. 5f,g and Extended Data Fig. 12b). As with extended treatment, there was no effect on *Lgr5, Axin2* or *Anxa1* expression, or proliferation/apoptosis in tumour epithelium following therapeutic challenge, and no clear impact transcriptionally (Extended Data Fig. 12c–i). To corroborate these results, epithelial response to treatment was examined via scRNA-seq of BPRNF tumours after 1 day or 3 days of treatment. This approach revealed no significant differences in any cell populations following treatment, in support of histological and bulk transcriptomic findings (Fig. 5h,i and Extended Data Fig. 12j,k). Despite the lack of epithelial-state changes, effect on proliferative or apoptosis-associated gene expression, a broad reduction in MAPK target gene expression was observed in single-cell transcriptomic datasets, suggesting effective target engagement in this model. These data suggest that RNF43 loss limits intrinsic tumour epithelial plasticity in response to MAPK-targeted therapies, and in doing so drives therapeutic response.

Long-term, durable treatment response is the major goal of managing advanced cancer. Current CRC-targeted therapies exhibit heterogeneity in both the initial response and duration of response, despite clinical use in genetically defined patient populations. Resistance to MAPK-targeting agents can occur through pathway reactivation, driven by mutation or amplification, and are neither readily actionable nor able to explain all cases of resistance^25,38–40^. The work presented here suggests that epithelial cellular plasticity is an adaptive response to therapy and acts as an important mechanism of resistance, which, to our knowledge, has not been previously described (Fig. 5j). Moreover, early metastatic disease exists in a vulnerable, restricted epithelial cell fate, which exhibits sensitivity to MAPK inhibition. These mechanisms may explain recent clinical trial observations including a recurrence-free survival benefit from the addition of panitumumab to neoadjuvant chemotherapy in EGFR ligand-enriched colon cancer, and the outstanding synergistic impact of first-line combined chemotherapy with BRAF + EGFR inhibition in metastatic *BRAF*-mutant CRC^41,42^. Restriction of epithelial cell populations to WNT-enriched states via MAPK inhibition may also provide an opportunity to augment or improve existing therapeutic modalities that directly impact the *Lgr5*^*+*^ stem population, such as cytotoxic chemotherapies, targeted radiotherapy or novel therapeutic agents^43,44^. Our data demonstrate that there is an incredible opportunity to improve outcomes for patients with CRC through sequential, cyclical or parallel targeting of multiple epithelial cell fates.

## Methods

### Mouse housing and ethics

All animal experiments were carried out according to the UK Home Office guidelines (project licences 70/8646, 70/9112 and PP3908577), with approval and oversight of the Animal Welfare and Ethics Review Board of the University of Glasgow. Mice were housed in conventional or individually ventilated cages at constant temperature (19–23 °C) and humidity (55 ± 10%) with a 12-h light–dark cycle. Mice were fed a standard chow diet and were given drinking water ad libitum. A mixture of individually ventilated cages and conventional open-top cages with environmental enrichment (tunnel and straw bedding) were used. No formal randomization or blinding was undertaken for in vivo experiments, with both female and male mice used.

### Mouse genetic alleles

The following alleles were utilized: VillinCre^ERT2^ transgene^45^, which results in expression of a tamoxifen-inducible *cre* recombinase in the intestinal epithelium; *Braf*^*LSL-V600E/+*^ (ref. ^46^) (hereafter and referred to as *Braf*^*V600E/+*^), which results in a mutant hyperactive BRAF protein; *Trp53*^*fl/fl*^ (ref. ^47^), which causes loss of p53 that emulates a loss-of-function mutation; *Rosa26*^*N1icd/+*^ (ref. ^48^), which is a transgene for the NOTCH1 intracellular domain protein causing the downstream effects of NOTCH1 activation; *Tgfbr1/Alk5*^*fl/fl*^ (ref. ^49^) (hereafter *Alk5*^*fl/fl*^), which causes loss of ALK5, emulating loss-of-function mutation of the TGFβ pathway; *Apc*^*fl*^ (ref. ^50^), which causes loss of APC that emulates a loss-of-function mutation; *Kras*^*LSL-G12D/+*^ (ref. ^51^) (hereafter and referred to as *Kras*^*G12D/+*^) or *Kras*^*LSL-G12C/+*^ (ref. ^52^) (hereafter and referred to as *Kras*^*G12C/+*^), which causes a mutant hyperactive KRAS protein; and *Rnf43*^*fl/fl*^ (ref. ^53^), which causes loss of RNF43 that emulates a loss-of-function mutation. Mice were genotyped using tissue taken during ear notching at the time of weaning and carried out by Transnetyx (Cordova TN) through established genotyping protocols. All mice were of a C57BL6/J background (≥N4). The description of generated mice is summarized in Extended Data Table 1.

### In vivo inductions

For all experiments, adult mice were induced at the mean (±s.d.) age of 12.7 weeks (±3.4 weeks). Genetically engineered mice were induced with a single 2 mg intraperitoneal injection of tamoxifen (T5648, Sigma-Aldrich) when mice weighed more than 20 g. Tamoxifen was dissolved in absolute ethanol to make a stock solution of 100 mg ml^−1^. This was diluted to a final working concentration of 10 mg ml^−1^ in corn oil for intraperitoneal injection (C8267, Sigma-Aldrich). Unless otherwise stated, mice were aged to clinical end points defined by a combination of objective measures related to intestinal tumour burden, including loss of body weight (no greater than 20%), altered posture, piloerection and anaemia (pallor). These limits were not exceeded in any experiment carried out during this study. APC and AKG12D/C models were induced via a single 70-μl endoscope-guided injection of 4-hydroxytamoxifen (100 μM in PBS) into the colonic submucosa, with the exception early time-point studies, which were induced with three 70-μl injections. Colonic tumour burden following local induction was confirmed by colonoscopy, with mice subsequently enrolled into treatment cohorts or sampled.

### In vivo drug treatments

All drug doses were calculated based on the weight of a 25 g mouse. Treatments were commenced at varying time points for different experiments as indicated. Unless otherwise stated, drugs were formulated in a suspension with 0.5% hydroxypropyl methylcellulose + 0.1% Tween-80 (hereafter hydroxypropyl methylcellulose vehicle) unless otherwise stated. BRAF inhibition was conducted with dabrafenib (D-5678, LC Labs) and administered at 30 mg kg^−1^ in 100 μl by oral gavage once daily continuously. EGFR inhibition was conducted by erlotinib (E-4997, LC Labs) and administered at 80 mg kg^−1^ in 100 μl by oral gavage once daily continuous dosing. MEK1/2 inhibition was conducted using AZD6244 (AstraZeneca) and administered at 25 mg kg^−1^ in 100 μl by oral gavage twice daily continuous dosing. KRAS(G12C) inhibition was conducted using AZD4625 (AstraZeneca), administered at 100 mg kg^−1^ by oral gavage once daily. For KRAS(G12C) inhibitor intracolonic time points, mice received four doses of AZD4625 and were euthanized following the fourth dose. KRAS(G12D) inhibition was performed using 7.5 mg kg^−1^ intraperitoneal injection of MRTX1133 (Mirati Therapeutics) twice daily in 100 μl of vehicle (10% hydroxypropyl β-cyclodextrin in citrate buffer pH 5). For intracolonic time points, mice received seven doses of MRTX1133 over 4 days and were euthanized following AM dose on the fourth day. For intrasplenic transplantations, treatments were commenced 7 days after transplantation and given continuously for 3 weeks. KRAS(G12D) inhibition in the intrasplenic experiments was performed with RMC9805 (HY-156819, MedChemExpress) and administered at 100 mg kg^−1^ in 250 μl vehicle (10% DMSO, 20% PEG400 (Sigma), 10% Solutol HS15 (HY-Y1893, MedChemExpress) and 60% water) by oral gavage once daily for 3 weeks. Pan-RAS(on) inhibition was conducted with RMC6236 (HY-148439, MedChemExpress) and administered at 25 mg kg^−1^ in 250 μl of vehicle (10% DMSO, 20% PEG400 (Sigma), 10% Solutol HS15 (HY-Y1893, MedChemExpress) and 60% water) by oral gavage once daily for 3 weeks.

### Intrasplenic liver metastasis transplantations

Mouse tumour-derived organoids were generated and maintained as previously described^22^. These were injected intrasplenically into male immune-competent C57BL/6J mice (Charles River strain 632). Male recipient mice were used in orthotopic transplantation models to correspond to organoid lines. Tumour-derived organoids were mechanically dissociated into fragments by pipetting and then washed twice in PBS. They were enzymatically digested using 0.25% trypsin in PBS–EDTA at 37 °C for 7 min (3 ml trypsin–PBS–EDTA per six-well plate). Ten per cent FBS in PBS was then added (12 ml per six-well plate) and the suspension was passed through a 40-μm cell strainer. The cells were then washed, pelleted and resuspended in 1 ml PBS and counted using the Countess automated cell counter (ThermoFisher). Approximately 500,000 single cells in 50 μl PBS were injected into each mouse. Mice were anaesthetized with isofluorane and a laparotomy was performed to gain access to the spleen where cells were injected. The abdominal wound was closed with absorbable sutures and the skin was closed with staples, which were removed 7 days post-procedure. Organoid lines for each experiment are listed in Extended Data Table 2, and were routinely confirmed mycoplasma free before transplantation. Details of the lines that underwent scRNA-seq are listed in Extended Data Table 3.

### Intracolonic transplantation

Mouse tumour-derived organoids were injected in the left colonic submucosa into male immune-competent C57BL/6J mice (Charles River strain 632) using previously described methods^54^. Male recipient mice were used in orthotopic transplantation models to correspond to organoid lines. Tumour organoids were mechanically dissociated into fragments by pipetting and washed twice in PBS. Each mouse was injected with the equivalent of one well from a six-well plate in 70 μl of PBS. This was injected into the colonic submucosa using a Karl Storz TELE PACK VET X LED endoscopic video unit with associated needle. Mice were monitored for tumour formation via colonoscopy and started treatment when tumour formation was confirmed. For AKPT transplants, this was 9 days after transplantation, and for KPN transplants, this was 16 days post-transplantation. Transplanted mice were aged to clinical end point. Organoid lines for each experiment are listed in Extended Data Table 4 and were routinely confirmed mycoplasma free before transplantation.

### Tissue sampling, fixation and staining

Mice were culled and dissected. The following were removed and placed in 10% neutral buffered formalin; mesenteric lymph nodes, liver, spleen, pancreas, lungs and any deposits of metastasis out with these organs. For genetically engineered mice induced by systemic, intraperitoneal injection of tamoxifen, the intestines were removed, flushed with water, opened longitudinally and pinned onto a wax disk with the lumen facing upwards. For intracolonic models, the tumour and normal adjacent tissue was excised, opened and fixed on Whatman paper. Tissue was fixed in 10% neutral buffered formalin for 24–72 h. Following fixation, intestines were rolled to generate a ‘Swiss roll’ and a 25-G needle placed through the middle to fix in place. The rolls were placed in 70% ethanol. Samples were processed and embedded in paraffin blocks by the CRUK Scotland Institute histology facility using standard techniques. Sections (3–4 μm) were cut for haematoxylin and eosin (H&E), immunohistochemistry (IHC) and in situ hybridization (ISH) staining techniques. H&E was stained using standard protocols. Each RNAscope run used *Dapb* as a negative control and *Ppib* as a positive control. RNAscope probes and antibodies are detailed in Extended Data Tables 5 and 6, respectively.

IHC-stained and ISH-stained slides were digitalized using a Leica SCN400F slide scanner at ×20. Images were analysed using HALO v2.0 (Indica Labs) image analysis software. Tumours of interest were manually annotated and stains were quantified and expressed as positive cells per tumour μm^2^ or positive probes per tumour μm^2^. The *H*-score was calculated using HALO v2.0. The *H*-score accurately represents staining intensity in IHC stains on a scale of 0–300 and is calculated as follows (1 × percentage of weak staining) +  (2 × percentage of moderate staining) + (3 × percentage of strong staining). For representative images of histology slides, figures were generated using scanned slides with HALO v2.0.

### FISH

Slides were prepared and stained according to the manufacturer’s recommendations for two channel ISH (ACD, RNAscope Multiplex Fluorescent Reagent Kit v2). Probe *Lgr5* (312171) was used for channel 1, whereas *Anxa1* (509291-C2) was used for channel 2. For intracolonic time-course studies, the RNAscope LS multiplex fluorescent reagent kit (322800) with 2.5 LS probe Mm-Anxa1 (509298) and 2.5 LS probe Mm-Lgr5-C2 (313178-C2) was used. Staining was performed on Leica Bond Rx autostainer strictly following the manufacturer’s instructions. Images were scanned at ×40 using Evident VS200 slides scanner. Images analysis was carried out using QuPath image analysis software. Areas of interest were annotated and the number of detections for each of the two stains was measured. The proportion of single-positive cells detected for each channel or stain, co-labelled with DAPI, was calculated in relation to the total number of detected cells.

### Tumour scoring

For small intestinal tumour models, after fixation on a wax plate before rolling, macroscopic intestinal tumour number and sizes were measured and recorded. For intracolonically induced models, tumour size was measured when put on Whatman paper. For intrasplenic liver metastasis models, tumour size and burden was based on macroscopic assessment at time of dissection.

### Xenium preparation and data capture

Formalin-fixed, paraffin-embedded blocks were sectioned on an automated microtome at 4 µm and placed onto microscope slides (MSS4511PK, Solmedia). Slides were stained with H&E using standard methods. H&E slides were scanned on the Aperio AT2 (Leica Microsystems) at ×40 magnification and images were uploaded to Concentriq (v3.7.4:95380 cd.). Each image was annotated with a region of interest (ROI) for Xenium analysis. Formalin-fixed, paraffin-embedded blocks were lightly scored with a microtome blade around the ROI. Workspaces were cleaned with RNaseZAP (R2020, Sigma-Aldrich), and blocks were rehydrated and sectioned at 4 µm. Up to five scored ROIs were individually placed onto Xenium slides (1000460, 10X Genomics) within the fiducial frame. A serial section was collected for H&E using the same tissue orientation. Slides were baked at 60 °C for 60 min and stored overnight at room temperature to ensure optimal tissue adhesion.

Slides were baked and dewaxed on the Ventana Discovery Ultra (Roche Tissue Diagnostics, RUO Discovery Universal v21.00.0019), and then placed in nuclease water for 20 s. Slides were dried and assembled into Xenium cassettes for manual staining. All heated manual staining steps were performed on a thermal cycler (846-x-070-241, Analytik Jena), and reagents were removed with a pipette between each protocol step. Extended Data Table 7 contains a description of reagents, and detailed panel description can be found in Supplementary Information.

Slides were incubated with 500 µl of decrosslinking buffer at 80 °C for 30 min and 22 °C for 10 min. Slides were washed with three changes of PBS-T for 1 min, then incubated with 500 µl of probe hybridization mix (lot 174243) at 50 °C for 16–24 h. Slides were washed with two changes of PBS-T for 1 min, then incubated with 500 µl post-hybridization wash buffer at 37 °C for 30 min. Slides were washed with three changes of PBS-T for 1 min, then incubated with 500 µl ligation mix at 37 °C for 2 h. Slides were washed with three changes of PBS-T for 1 min, then incubated with 500 µl amplification master mix at 30 °C for 2 h. Slides were washed with three changes of TE buffer for 1 min, then incubated with 500 µl of diluted reducing agent B for 10 min. Slides were washed with three changes of 70% ethanol for 1 min, followed by 500 μl autofluorescence solution for 10 min in the dark, and then washed in three changes of 100% ethanol for 2 min. Slides were dried at 37 °C without cassette lid and without closing the thermal cycler lid for 5 min. Slides were washed with 1,000 μl of PBS for 1 min in the dark, followed by 1,000 μl PBS-T for 2 min in the dark. Of Xenium nuclei staining buffer, 500 µl was applied for 1 min in the dark. Slides were washed in three changes of PBS-T for 1 min in the dark, then 1,000 μl PBS-T was added and slides were transferred to the Xenium analyser (v3.1.0.0). Decoding reagents and consumables were loaded onto the analyser, samples were annotated for analysis and the imaging and decoding run was started.

The automated quality control report generated by the Xenium analyser was reviewed upon run completion. Cell segmentation and instrument performance were manually verified in Xenium Explorer (v3.1.1) before data analysis.

### Xenium data analysis and visualization

Using the DAPI staining-derived nuclear segmentation, Proseg v2.0.4 (https://github.com/dcjones/proseg)^55^ was used to assign transcripts to cells and generate a counts table. In data relating to Fig. 3, four genes were removed from the analysis to account for panel differences between two Xenium runs (see Supplementary Information). Cells with less than 10 counts were discarded using scanpy.pp.filter_cells(min_counts=10) (scanpy v1.11.2). Data was log_1_*P* transformed using scanpy.pp.log1p using standard parameters. PCA was performed using scanpy.pp.pca (50 PCs). Neighbourhood graph was calculated using scanpy.pp.neighbors using default parameters. scanpy.tl.umap was used for UMAP projection of the data. Leiden clustering was performed using scanpy.tl.leiden at various resolutions depending on the dataset (resolution = 0.8 for Extended Data Fig. 4a and resolution = 1.2 for Extended Data Fig. 6a). The score_genes function in scanpy was used to calculate gene scores (default parameters, except for n_bins = 5, ctrl_size = 20). Commonly used Python libraries (Python v3.11.13, matplotlib v3.10, Seaborn v0.13, numpy v2.2.6, pandas v2.3.1, scipy v1.16.0, anndata v0.11.4 and shapely v2.1.1) were applied to visualize spatial distribution of cells. Cell-type probability vectors for each cell were generated by softmax-transforming score_genes outputs. For PCA analysis and visualization, probability vectors were centred, log-ratio transformed and used as input to sklearn.decomposition.PCA (scikit-learn v1.7.2). For metastasis size versus complexity analysis, individual liver metastases from AKPT intrasplenic organoid transplant mice (*n* = 3) were outlined based on H&E morphology, and coordinates were exported using Xenium Explorer v4. Tumour epithelial cells were subset, and the percentage of cells in the largest Leiden cluster was calculated for each metastasis. Pearson’s *r* was calculated to summarize the overall association between metastasis size and tumour heterogeneity. To test for trends between size and tumour heterogeneity, several regression models were fitted, and the best model was chosen based on the lowest Akaike’s information criterion. Model fit was quantified using the coefficient of determination (*R*^2^).

### Bulk RNA-seq

For tissues, pieces of tumour or intestinal tissue were sampled from mice, placed in RNAlater (R0901, Sigma-Aldrich) and stored at −80 °C before extraction. Workspaces were cleaned with RNaseZAP (R2020, Sigma-Aldrich). RNA was extracted from samples using the RNeasy Mini Kit (74104, Qiagen) following the manufacturer’s instructions. For tissues, homogenization was done by using Precellys ceramic bead-filled tubes in a Precellys Evolution machine (Bertin Instruments). For organoid pellets, cells were dissociated using pipetting in the first step of the extraction protocol. Final RNA elution was done in 30 µl of RNase-free water and RNA concentration was quantified using a NanoDrop 2000c (Thermo Scientific).

RNA quality was assessed by TapeStation and only samples with RNA integrity number ≥ 7 were sequenced. Library preparation and sequencing were done via an external commercial company, GENEWIZ (Azenta Life sciences), using an Illumina mRNA polyA selection library preparation kit and a Novaseq sequencer, sequencing 2 × 150 bp to a median depth of 23 million reads per sample. Sequences were of high quality with more than 90% bases having a quality score of at least 30. Sequences were aligned to mouse genome build GRCm38.98 (mm10) using Hisat2 (v2.1.0), and per gene counts were determined using FeatureCounts (v1.6.4). Analysis was performed in R (v4.5.1). Duplicated genes and genes with a total read count below 10 across 20–25% of the total number of samples were removed. Batch correction was performed in R using the Combat_seq function from the sva package (v3.56.0). All raw count matrices were normalized using DESeq2 (v1.42.1 or v1.48.0). DESeq2 was run on each genotype separately (when investigating effects of treatments), with the exception of analysis that was comparing genotypes in the absence of treatment. The design matrix was only ever one variable in this case (for example, genotype or treatment). The data were transformed using variance stabilization transformation, with blind = false. The intestinal SCI (v1.1.0)^22^ was applied to the variance stabilization transformation matrices, using geneid = ‘genename’ and organism = ‘mouse’. Single-sample gene set enrichment analysis was carried out using GSVA (v1.50.5). Boxplots were created using ggplot2 (v3.5.1 or v3.5.2) and ggbeeswarm (v0.7.2) with statistical annotation created by ggpubr (v0.6.0), method = ‘t-test’. Heatmaps were created using ComplexHeatmap (v2.18.0 or v2.20.0) and circlize (v0.4.16). The Supplementary Information contains gene lists and associated primary references of gene sets uses in this article.

### scRNA-seq

For the APC, AKG12D, AKG12D + treatment series, the APC, BP, BPRNF untreated series and the APC, BPNA, BPNA + treatment series, colonic tumour samples were collected in PBS and processed immediately for scRNA-seq. In brief, samples were chopped to achieve a paste-like consistency using a Mcllwain tissue chopper. Samples were transferred to GentleMACS C tubes (130-093-237, Miltenyi Biotec) for tissue homogenization using the GentleMACS Octo Dissociator with heaters (130-096-427, Miltenyi Biotec) in combination with the mouse tumour dissociation kit (130-096-730, Miltenyi Biotec). Following tissue digestion, samples were resuspended in RPMI + 10% FBS + 2 mM EDTA and filtered through 70-μm and 40-μm strainers, respectively. The cell suspension was centrifuged at 400*g* for 3 min and washed with RPMI + 0.4% BSA. The sample suspensions were centrifuged at 400*g* for 3 min and the resultant pellets were resuspended in 2% FCS + 25 mM HEPES + 2 mM EDTA + PBS for cell sorting. Cells were sorted using a BD FACSAria sorter (BD Biosciences) and DAPI (D1306, Invitrogen) to remove dead cells.

Live-sorted single cells were loaded onto a Chromium Chip G using reagents from the 10X Chromium Single-Cell 3′ v3 Gel Bead Kit and Library (10X Genomics) according to the manufacturer’s protocol. Libraries were analysed using the Bioanalyzer High Sensitivity DNA Kit (Agilent Technologies) and sequenced on the Illumina Novaseq 6000 with paired-end 150-base reads. Sequence alignment of single-cell data to the mm10 genome was performed using the count tool from the Cellranger package (v6.1.2) according to the developers’ instructions. Subsequent analysis was performed using R software (v4.5.1) and Seurat package (v5.2.1). Samples were input using the Read10X function, filtering to include cells with a minimum of 100 expressed genes and genes that are present in at least three cells. Samples were further filtered to only include cells with less than 10% mitochondrial genes, less than 10% haemoglobin genes, more than 100 genes per cell and more than 400 reads per cell. Individual biological replicates with less than 100 cells passing quality control were removed before clustering. Samples were then integrated by RPCA using the IntegrateData function before being scaled, regressing out the number of features, and normalized. Dimension reduction was then performed using PCA before clustering was performed using the FindNeighbours and FindClusters functions. Marker genes for individual clusters were determined using the FindAllMarkers function. Major cell types were annotated using scGate (v1.7.0) and then epithelial subtypes were annotated using custom gene lists described in the Supplementary Information. Gene set scores are calculated using AddModulescore in Seurat. To identify the cell-type signature from scRNA-seq, the FindAllMarker function in Seurat was used with these parameters (log_2_fold change > log_2_(1.5), adjusted *P* < 0.05 and percentage of expressing cells in target cell type > 10%). The SCI score for scRNA-seq was calculated as the difference of two module scores (RSC − CBC).

For the AKG12C, APC and BPRNF treatment series, tumours were processed to single cells as follows using GEM-X Flex kit (GEM-X Flex Sample Preparation 10000781) and Protocols by 10X Genomics (https://www.10xgenomics.com/products/flex-gene-expression); tissues were weighed and minced in a glass petri dish and incubated overnight in fixation buffer B (with nuclease-free H_2_0 and 4% formaldehyde) overnight at 4 °C. Samples were centrifuged at 1,000 rcf × 5 min at 4 °C, and supernatant was removed and washed in PBS. The spin was repeated, supernatant aspirated and samples were resuspended in quench buffer. Quench buffer was removed and dissociation solution of RPMI media, and Liberase TH 5 mg ml^−1^ (5401135001, Millipore Sigma) was added and samples were transferred to a gentleMACS C tube (130-093-237, Miltenyi Biotec) for tissue homogenization on the GentleMACS Octo Dissociator with heaters (130-096-427, Miltenyi Biotec). The homogenized tissue was filtered through a 30-µm strainer (130-098-458, MACS SmartStrainer 30 µm) and centrifuged at 850 rcf × 5 min. Supernatant was removed and cells were resuspended in 1 ml of quench buffer before counting using equal parts cell suspension and ReadyCount Green/Red Viability Stain (A49905, Invitrogen) on the CellDrop Automated Cell Counter (DeNovix). AKPT SIT, KPN SIT and KPN LMET organoids were processed to a single-cell suspension as follows. Organoids were harvested 72 h after seeding and incubated in Cell Recovery Solution (354253, Corning) on ice for 20 min to remove Matrigel. Samples were centrifuged at 300*g* for 5 min at 4 °C, and supernatant was removed and resuspended in 1 ml PBS and mechanically dissociated. This spin was repeated and supernatant was removed. Samples were resuspended and incubated in 1 ml TrypLE Express (12604013, Gibco) supplemented with 10 µM Y27632 (ROCKi) (G9145, Sigma-Aldrich) for 15 min at 37 °C. The digestion was inhibited by addition of 10 ml ADMEM + 10%FBS and cells were filtered through a 40-µm strainer. The single-cell suspensions were centrifuged at 300*g* for 5 min at 4 °C and supernatant was removed. Cells were resuspended and incubated in fixation buffer B overnight at 4 °C. Organoid samples were centrifuged at 1,000 rcf for 5 min at 4 °C, and supernatant was removed and washed in PBS. This step was repeated, supernatant aspirated and samples were resuspended in quench buffer before cell counting as per the tissue-processing protocol.

Cell input was normalized across all samples, with each sample hybridized with a unique probe barcode using the GEM-X Flex Mouse Transcriptome Probe Kit (PN-1000786), according to protocols by 10X Genomics. Following hybridization, samples were pooled and washed using the pooled wash method, with cells further counted and diluted to a density sufficient to capture 10,000 cells per sample and processed through the Chromium X controller to generate multiplexed barcoded gel-beads in emulsion (GEMs). The GEMs were then processed through various ligation and extension steps to add unique molecular identifiers (UMIs), GEM barcodes and partial read 1 primer. The GEMs were then broken, with a round of pre-amplification before final library preparation in which full-length indexed libraries containing the Illumina P5 and P7 sequencing sites were generated. Complete library fragments containing GEM cell barcodes, UMIs and ligated probe inserts were then sequenced to a depth of approximately 100 million reads per sample (10,000 reads per cell) on an Illumina NextSeq 2000 benchtop sequencing platform.

After sequencing, multiplexed libraries were demultiplexed, UMIs counted and count tables produced, using the Cellranger 9.0.0 ‘multi’ function and a modified Chromium_Mouse_Transcriptome_Probe_Set_v1.1.1_GRCm39-2024 annotation set. Sample-level data were then integrated, filtered and annotated in Seurat v5.2.1 as described above.

### Gene lists

The list of gene sets used in analysis of bulk, single-cell and spatial transcriptomics can be found in the Supplementary Information.

### Statistics

Data were analysed by statistical tests (described in the figure legends) using Graphpad Prism v10, with the exception of the RNA-seq data, as described above. Two-sided tests were carried out as default unless otherwise stated. For normally distributed data, parametric tests were used, and non-parametric tests were used for non-normal data. In all cases where *t*-test is mentioned, this refers to a Student’s *t*-test. Proportions were compared using the Fisher’s exact test. Survival analysis was performed using the Kaplan–Meier method and compared with the log-rank test. Results were considered significant when *P* < 0.05. Some diagrams and pictorial schemas were generated using BioRender (https://www.biorender.com) and have associated publication licences.

### Reporting summary

Further information on research design is available in the Nature Portfolio Reporting Summary linked to this article.

## Online content

Any methods, additional references, Nature Portfolio reporting summaries, source data, extended data, supplementary information, acknowledgements, peer review information; details of author contributions and competing interests; and statements of data and code availability are available at 10.1038/s41586-025-09916-w.

## Supplementary information

Supplementary Information This file contains gene lists used throughout this study for module scoring and gene enrichment analysis, and details of the Xenium spatial transcriptomics panel used.

Reporting Summary

Supplementary Information Table of Contents.

## Figures and Tables

**Extended Data Table 1:**
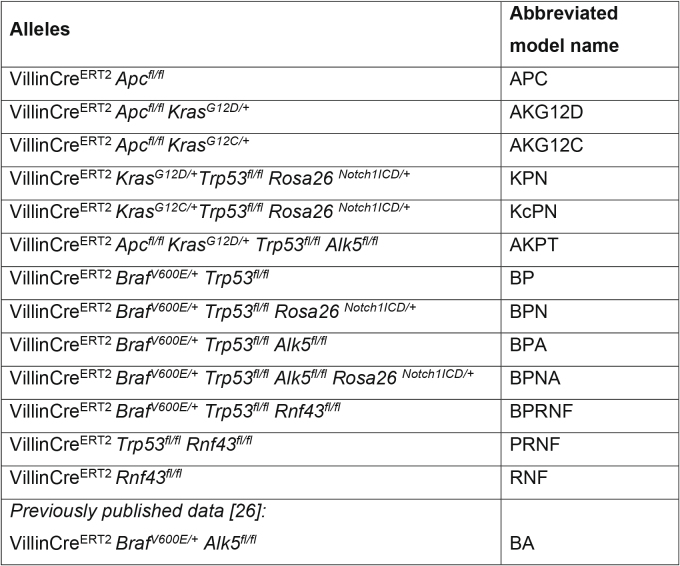
Summary of mouse genotypes

**Extended Data Table 2:**
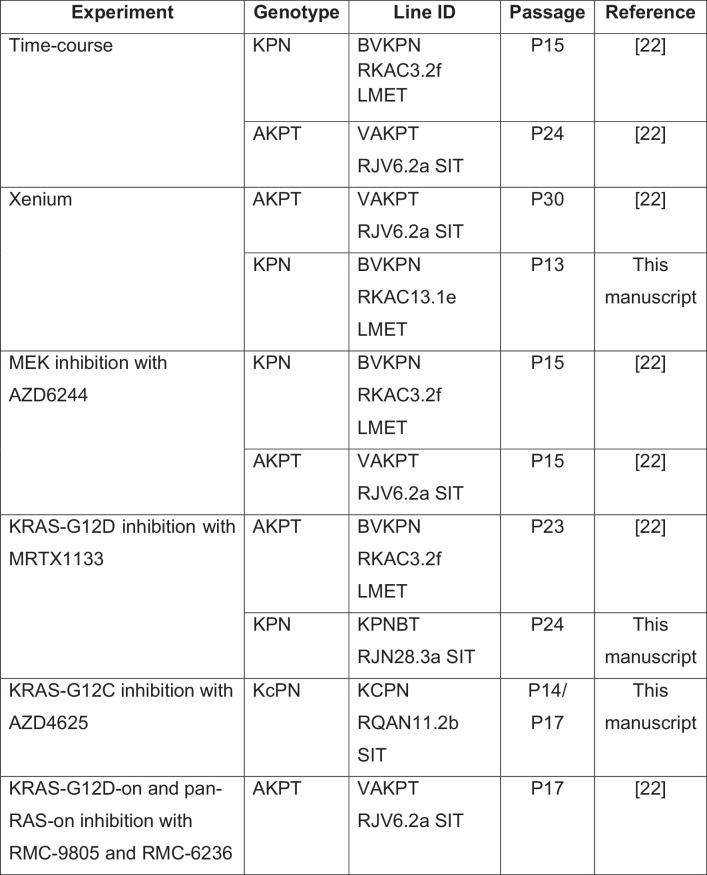
Intrasplenic transplantation organoid line description (SIT=Small Intestinal Tumour line, LMET=Liver Metastasis Line)

**Extended Data Table 3:**
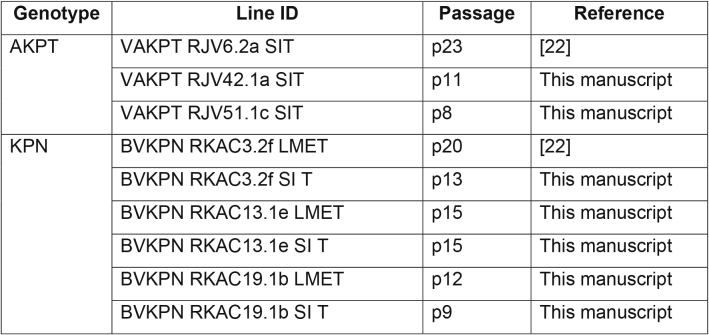
Details of organoid lines that underwent single cell RNA sequencing

**Extended Data Table 4:**
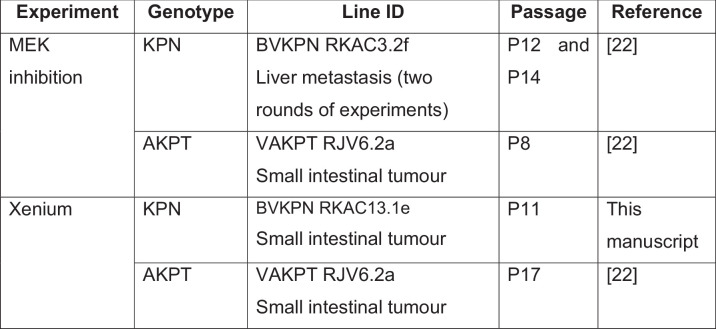
Description of intracolonically tranplanted lines

**Extended Data Table 5:**
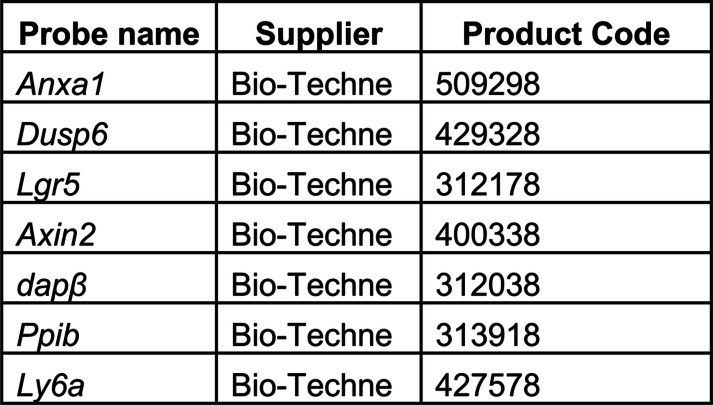
Details of RNAscope probes used

**Extended Data Table 6:**
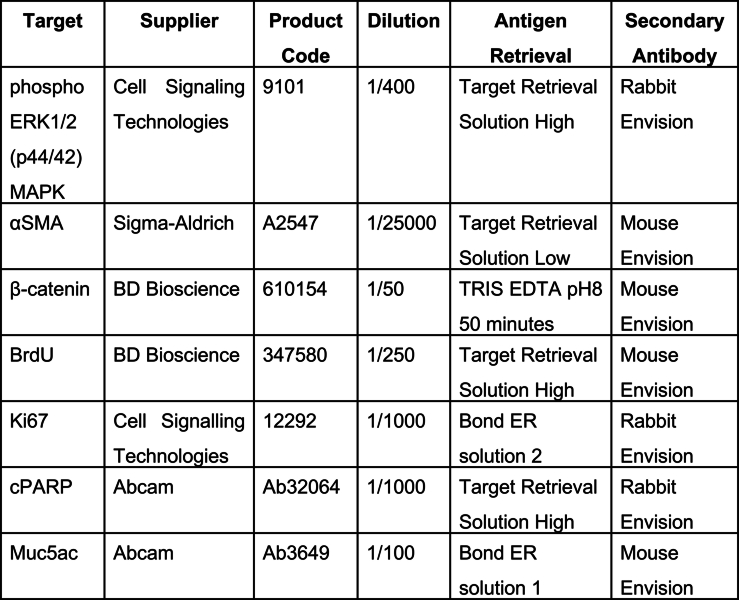
Details of IHC antibodies used

**Extended Data Table 7:**
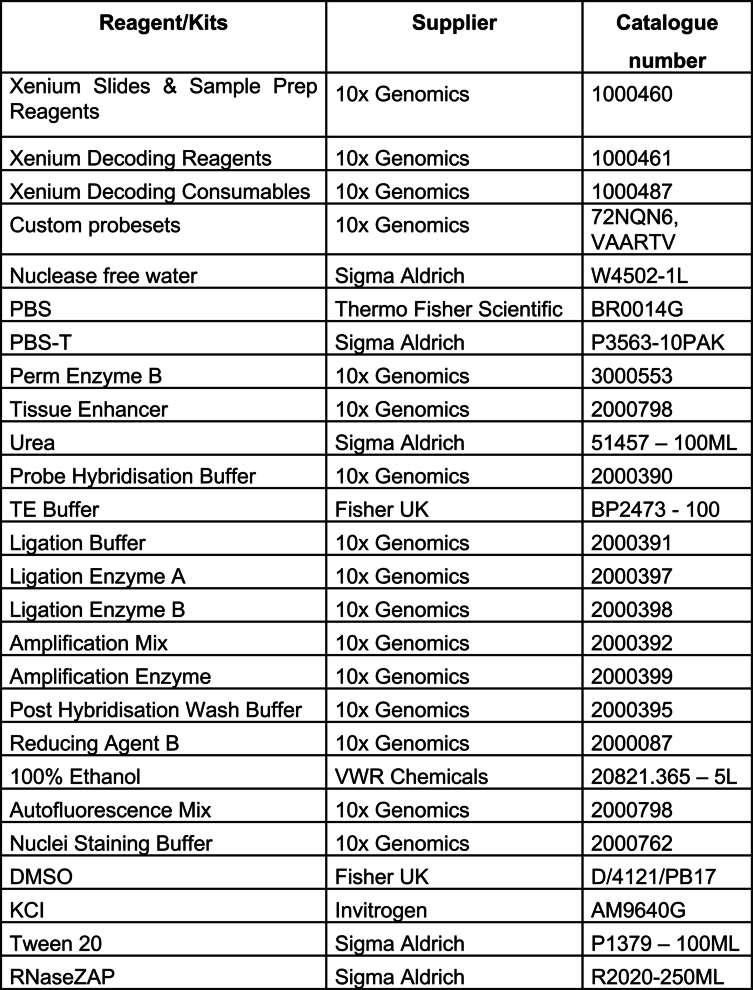
Xenium Reagents

## Data Availability

Bulk RNA-seq data used in this article are available through the Gene Expression Omnibus (https://www.ncbi.nlm.nih.gov/geo/) under the accession number GSE307773. scRNA-seq data are available through the Gene Expression Omnibus under accession numbers GSE307774, GSE308102, GSE308125, GSE308130 and GSE308133. scRNA-seq and Xenium-processed objects are available via Zenodo^56,57^ (10.5281/zenodo.17106157 and 10.5281/zenodo.17414559). The reference mouse genome assembly GRCm38.98 (mm10; https://ftp.ensembl.org/pub/release-98/gtf/mus_musculus/) was used for sequence alignments. All other data are available from the corresponding authors on reasonable request.
