# Supplementary material for: MAPK-driven epithelial cell plasticity drives colorectal cancer therapeutic resistance
Source: Nature. 2025 Nov 24;650(8102):748–58. doi: 10.1038/s41586-025-09916-w (PMC12916511; doi:10.1038/s41586-025-09916-w)
Supplement: Supplementary file 2 — Reporting Summary [file 41586_2025_9916_MOESM2_ESM.pdf]

nature portfolio

Corresponding author(s): Andrew D. Campbell & Owen J. Sansom

Last updated by author(s): Oct 21, 2025

Reporting Summary

Nature Portfolio wishes to improve the reproducibility of the work that we publish. This form provides structure for consistency and transparency in reporting. For further information on Nature Portfolio policies, see our Editorial Policies and the Editorial Policy Checklist.

Statistics

For all statistical analyses, confirm that the following items are present in the figure legend, table legend, main text, or Methods section.

n/a	Confirmed	
☐	☒	The exact sample size (*n*) for each experimental group/condition, given as a discrete number and unit of measurement
☐	☒	A statement on whether measurements were taken from distinct samples or whether the same sample was measured repeatedly
☐	☒	The statistical test(s) used AND whether they are one- or two-sided<br>*Only common tests should be described solely by name; describe more complex techniques in the Methods section.*
☒	☐	A description of all covariates tested
☐	☒	A description of any assumptions or corrections, such as tests of normality and adjustment for multiple comparisons
☐	☒	A full description of the statistical parameters including central tendency (e.g. means) or other basic estimates (e.g. regression coefficient) AND variation (e.g. standard deviation) or associated estimates of uncertainty (e.g. confidence intervals)
☐	☒	For null hypothesis testing, the test statistic (e.g. *F*, *t*, *r*) with confidence intervals, effect sizes, degrees of freedom and *P* value noted<br>*Give P values as exact values whenever suitable.*
☒	☐	For Bayesian analysis, information on the choice of priors and Markov chain Monte Carlo settings
☒	☐	For hierarchical and complex designs, identification of the appropriate level for tests and full reporting of outcomes
☒	☐	Estimates of effect sizes (e.g. Cohen's *d*, Pearson's *r*), indicating how they were calculated

Our web collection on statistics for biologists contains articles on many of the points above.

Software and code

Policy information about availability of computer code

Data collection	No external datasets were used. No custom code was used. All R packages used for analysis are included in the methods section.
Data analysis	Bulk RNA-seq analysis software: HISAT2 v2.1.0, FeatureCounts v1.6.4, R v4.5.1, sva v3.56.0, DESeq2 v1.42.1 and v1.48.0, ISCindex v1.1.0, ggplot2 v3.5.1, ggbeeswarm v0.7.2, ggpubr v0.6.0, ComplexHeatmap v2.18.0 and v2.20.0, circlize v0.4.16.<br><br>scRNA-seq analysis software: Cell Ranger v6.1.2 (3' scRNA-seq) and Cell Ranger v9.0.0 (GEM-X Flex scRNA-seq), R v4.5.1, Seurat v5.2.1, scGate v1.7.0.<br><br>Xenium analysis software: Xenium Explorer v3.1.0, v3.1.1 and v4, Proseg v2.0.4, python 3.11.13, Scanpy v1.11.2, , matplotlib v3.10, seaborn v0.13, numpy v2.2.6, pandas v2.3.1, scipy1.16.0, anndata v0.11.4, shapely v2.1.1<br><br>Statistical analysis was done using Graphpad Prism V10 or R. IHC and ISH stained slides were digitalised using a Leica SCN400F slide scanner at 20X. Images were analysed using HALO v2.0 (Indica Labs) image analysis software. FISH slides were scanned at 40x using Evident VS200 slides scanner and analyzed on Qupath image analysis software v0.5.1.

For manuscripts utilizing custom algorithms or software that are central to the research but not yet described in published literature, software must be made available to editors and reviewers. We strongly encourage code deposition in a community repository (e.g. GitHub). See the Nature Portfolio guidelines for submitting code & software for further information.

Data

Policy information about availability of data

All manuscripts must include a data availability statement. This statement should provide the following information, where applicable:

- Accession codes, unique identifiers, or web links for publicly available datasets
- A description of any restrictions on data availability
- For clinical datasets or third party data, please ensure that the statement adheres to our policy

Bulk RNA sequencing data used in this manuscript are available through the Gene Expression Omnibus under accession number GSE307773. Single cell RNA sequencing data are available through the Gene Expression Omnibus (https://www.ncbi.nlm.nih.gov/geo/) under accession numbers GSE307774, GSE308102, GSE308125, GSE308130 and GSE308133. Single cell RNA sequencing processed objects are available via Zenodo accessible at DOI: 10.5281/zenodo.17106157. Reference murine genome assembly GRCm38.98 (mm10) (https://ftp.ensembl.org/pub/release-98/gtf/mus_musculus/) was used for sequence alignments. All other data are available from the corresponding authors on reasonable request. Xenium processed objects are available via Zenodo accessible at DOI: 10.5281/zenodo.17414559

Research involving human participants, their data, or biological material

Policy information about studies with human participants or human data. See also policy information about sex, gender (identity/presentation), and sexual orientation and race, ethnicity and racism.

Reporting on sex and gender	N/A
Reporting on race, ethnicity, or other socially relevant groupings	N/A
Population characteristics	N/A
Recruitment	N/A
Ethics oversight	N/A

Note that full information on the approval of the study protocol must also be provided in the manuscript.

Field-specific reporting

Please select the one below that is the best fit for your research. If you are not sure, read the appropriate sections before making your selection.

☒ Life sciences ☐ Behavioural & social sciences ☐ Ecological, evolutionary & environmental sciences

For a reference copy of the document with all sections, see nature.com/documents/nr-reporting-summary-flat.pdf

Life sciences study design

All studies must disclose on these points even when the disclosure is negative.

Sample size	All in vivo experiments were carried out in line with the 3R principles of replacement, reduction and refinement. Cohort sizes were based on initial effect sizes and SD from small pilots and then calculated using G*Power v3.1.9.6 (HHU Dusseldorf), using alpha and beta error values of 0.05 and 0.2 respectively.
Data exclusions	No data was excluded. Mice were censored, but data shown, if sampled for reasons unrelated to the variable under study.
Replication	For in vivo experiments, a minimum of 3 mice were used for experiments. For RNA seq, a minimum of 3 tumours from separate mice were used. For histological analysis a minimum of three different mice tumours were used for IHC and ISH. All replicates were considered successful.
Randomization	No formal randomization method was used for treatment experiments in GEMM mice due to the nature of breeding. To minimise genetic variability, all experimental and control animals were either generated on a pure, inbred genetic background, or from individual breeding colonies.
Blinding	For animal welfare reasons, no blinding occurred in the experimental/data collection phase. During histological analysis, researchers were blinded to genotype/treatment groups.

Reporting for specific materials, systems and methods

We require information from authors about some types of materials, experimental systems and methods used in many studies. Here, indicate whether each material, system or method listed is relevant to your study. If you are not sure if a list item applies to your research, read the appropriate section before selecting a response.

Materials & experimental systems

n/a	Involved in the study	
☐	☒	Antibodies
☒	☐	Eukaryotic cell lines
☒	☐	Palaeontology and archaeology
☐	☒	Animals and other organisms
☒	☐	Clinical data
☒	☐	Dual use research of concern
☒	☐	Plants

Methods

n/a	Involved in the study	
☒	☐	ChIP-seq
☒	☐	Flow cytometry
☒	☐	MRI-based neuroimaging

Antibodies

Antibodies used

RNAScopes:
Anxa1; Bio-Techne; Cat#509298
Dusp6; Bio-Techne; Cat#429328
Lgr5; Bio-Techne; Cat#312178
Axin2; Bio-Techne; Cat#400338
Ly6a; Bio-Techne; Cat#427578
dapβ; Bio-Techne; Cat#312038
PPIB; Bio-Techne; Cat#313918

Antibodies:
phospho ERK1/2 (p44/42) MAPK; Cell Signaling Technology; Cat#9101; Dilution 1/400; Antigen retrieval: Target Retrieval Solution High; Secondary antibody - Rabbit Envision
αSMA; Sigma-Aldrich; Cat#A2547; Dilution 1/25000; Antigen retrieval: Target Retrieval Solution Low; Secondary antibody - Mouse Envision
β-catenin; BD Bioscience; Cat#610154; Dilution 1/50; Antigen retrieval: TRIS EDTA pH8 50 minutes; Secondary antibody - Mouse Envision
BrdU; BD Bioscience; Cat#347580; Dilution 1/250; Antigen retrieval: Target Retrieval Solution High; Secondary antibody - Mouse Envision
Muc5ac; Abcam; Cat#Ab3649; Dilution 1/100; Antigen retrieval: Bond ER solution 1; Secondary antibody - Mouse Envision
Ki67 (D3B5); Cell Signalling Technologies; Cat#12292; Dilution 1/1000; Antigen retrieval: Bond ER solution 2; Secondary antibody - Rabbit Envision
cleavedPARP; Abcam; Cat#Ab32064; Dilution 1/1000; Antigen retrieval: Target Retrieval Solution High; Secondary antibody - Rabbit Envision

Validation

All antibodies and in-situ probes were commercial sourced and selected based upon manufacturers recommendations regarding the target species. All antibodies and in-situ probes were optimized and validated using a suitable control tissue with a known expression of the target of interest. The location of antibody binding and probe hybridization and intensity of signal produced were evaluated under validation.

Animals and other research organisms

Policy information about studies involving animals; ARRIVE guidelines recommended for reporting animal research, and Sex and Gender in Research

Laboratory animals

All mice were of a C57BL6 background (≥N4). Mice were housed in conventional cages at constant temperature (19–23°C) and humidity (55 ± 10%) with a 12-hour light/dark cycle. Mice were fed a standard chow diet and were given drinking water ad libitum. A mixture of individually ventilated cages and conventional open-top cages with environmental enrichment (tunnel and straw bedding) were used. Genetically engineered mice were induced at a mean age of 12.7 weeks (SD+/-3.4 weeks).

Wild animals

None

Reporting on sex

As intestinal cancer affects people of both sex, analysis was not split up by sex. Both female and male mice were used for experiments, with the exception of recipient mice in orthotopic transplantation experiments. Recipient mice and donor organoid lines sex matched, with only male derived lines used.

Field-collected samples

None

Ethics oversight

All animal experiments were carried out according to the UK Home Office guidelines (project licences 70/8646, 70/9112 and PP3908577), with approval and oversight of the Animal Welfare and Ethics Review Board (AWERB) of the University of Glasgow.

Note that full information on the approval of the study protocol must also be provided in the manuscript.

Plants

Seed stocks	NA
Novel plant genotypes	NA
Authentication	NA
