# Supplementary material for: MAPK-driven epithelial cell plasticity drives colorectal cancer therapeutic resistance
Source: Nature. 2025 Nov 24;650(8102):748–58. doi: 10.1038/s41586-025-09916-w (PMC12916511; doi:10.1038/s41586-025-09916-w)
Supplement: Supplementary file 3 — Table of Contents. [file 41586_2025_9916_MOESM3_ESM.docx]

Supplementary Information

This file contains gene lists used throughout this study for module scoring and gene enrichment analysis, and details of the Xenium spatial transcriptomics panel used.

| **Name in manuscript** | **Tab name** | **Description** | **Reference** |
| --- | --- | --- | --- |
| RSC | RSC | Genelist related to RSC score | <https://doi.org/10.1016/j.stem.2022.07.008> |
| CBC | CBC | Genelist related to CBC score | <https://doi.org/10.1016/j.stem.2022.07.008> |
| Sansom_Wnt_signature | WNT | Genelist for WNT activation | <https://doi.org/10.1101/gad.287404> |
| Apc_adenoma | Apc_Adenoma | Genelist of most differentially expressed genes between APC-deficient adenomas and WT tissue | This manuscript |
| Dry_MAPK | MAPK | Genelist for MAPK activation | <https://doi.org/10.1158/0008-5472.CAN-09-1577> |
| Dry_MEK_Inhib | MEK_Inhib | Genelist for MAPK inhibition | <https://doi.org/10.1158/0008-5472.CAN-09-1577> |
| Gregorieff_YAP | YAP | Genelist for YAP activation | <https://doi.org/10.1038/nature15382> |
| Foetal_Mustata | Fetal | Genelist for fetal spheroid cultures | <https://doi.org/10.1016/j.celrep.2013.09.005> |
| Xenium genelists – cell fate | Xenium_genelists | Genelists derived from scRNAseq analysis, then applied to Xenium datasets to define cell fates | This manuscript |
| Xenium full panel | 100 Gene Xenium Panel – 72NQN6 | Primary Xenium panel – 100 gene, murine | This manuscript |
|  | 100 Gene Xenium Panel - VAARTV | Secondary Xenium panel – 100 gene, murine | This manuscript |
